# Evodiamine inhibits both stem cell and non-stem-cell populations in human cancer cells by targeting heat shock protein 70

**DOI:** 10.7150/thno.49876

**Published:** 2021-01-01

**Authors:** Seung Yeob Hyun, Huong Thuy Le, Hye-Young Min, Honglan Pei, Yijae Lim, Injae Song, Yen T. K. Nguyen, Suckchang Hong, Byung Woo Han, Ho-Young Lee

**Affiliations:** 1 Creative Research Initiative Center for concurrent control of emphysema and lung cancer, College of Pharmacy, Seoul National University, Seoul 08826, Republic of Korea; 2 College of Pharmacy and Research Institute of Pharmaceutical Sciences, Seoul National University, Seoul 08826, Republic of Korea

**Keywords:** heat shock protein 70, evodiamine, cancer stem cells, antitumor, alkaloid

## Abstract

**Rationale:** Cancer stem cells (CSCs) are known to cause tumor recurrence and drug resistance. The heat shock protein (HSP) system plays a major role in preserving expression and function of numerous oncoproteins, including those involved in the CSC activities. We explored novel anticancer drugs, especially those targeting HSP components required for the functional role of CSCs.

**Methods:** Investigation of the role of the HSP system in CSCs and screening of a natural product chemical library were performed by utilizing cancer cell lines, primary cultures of patient-derived xenografts (PDXs), and their putative CSC subpopulations (i.e., those grown under sphere-forming conditions, stably transfected with reporter vectors carrying *NANOG* or *POUSF1* promoters, or carrying high ALDH activity) in vitro and PDX and *Kras*^G12D/+^-driven tumor models in vivo. Regulation of the HSP system was investigated by immunoprecipitation, drug affinity responsive target stability assay, binding experiments using ATP-agarose beads and biotinylated drug, and docking analysis.

**Results:** The HSP system was activated in CSCs via transcriptional upregulation of the HSP system components, especially HSP70. Evodiamine (Evo) was identified to induce apoptosis in both CSC and bulk non-CSC populations in human lung, colon, and breast cancer cells and their sublines with chemoresistance. Evo administration decreased the multiplicity, volume, and load of lung tumors in *Kras*^G12D/+^ transgenic mice and the growth of cancer cell line- and PDX-derived tumors without detectable toxicity. Mechanistically, Evo disrupted the HSP system by binding the N-terminal ATP-binding pocket of HSP70 and causing its ubiquitin-mediated degradation.

**Conclusions:** Our findings illustrate HSP70 as a potential target for eliminating CSCs and Evo as an effective HSP70-targeting anticancer drug eradicating both CSCs and non-CSCs with a minimal toxicity.

## Introduction

Cancer stem cells (CSCs), a rare population in tumors carrying stem cell-like properties 1, 2, are proposed to originate from normal stem cells (SCs) after either the sequential acquisition of genetic/epigenetic changes or the dedifferentiation of transit-amplifying/progenitor cells carrying genetic mutations 1, 3. CSCs have the ability to give rise to heterogeneous tumor cell population, playing important roles in tumor initiation, recurrence, metastasis, and anticancer drug resistance 1, 4. Therefore, development of anti-CSC therapeutic drugs could be a logical strategy for eradicating tumors. Several clinical trials targeting CSCs and their microenvironmental niches are ongoing 2, 3. However, the biology underlying the evolution and maintenance of CSCs remains obscure mainly due to the heterogeneous nature of CSCs and the lack of specific and reliable markers to identify CSCs 4, 5, especially those in non-small cell lung cancer (NSCLC), one of the most common types of cancer with the greatest incidence and mortality worldwide 6, 7. Consequently, there is no clinically available drug that inhibit CSCs.

The heat shock protein (Hsp) system, consisting of HSP90, HSP70, and their associated chaperone proteins, controls the conformational maturation and stability of numerous proteins, including oncoproteins that have been implicated in signaling networks associated with the hallmarks of cancer 8-10. Among the family, the HSP90 and HSP70 are the most studied families of HSPs 11. HSP90 constitutes multichaperone complexes with other chaperone proteins, including HSP70 12, playing a critical role in stabilization of several client oncoproteins that have been implicated in proliferation, survival, and resistance to anticancer drugs 12. HSP90 and HSP70 are frequently deregulated in a variety of tumor types and contribute to poor prognosis of cancer patients 10, 13, 14. Studies have shown elevated expression of some HSP-related genes in murine and human embryonic SCs 15-17, indicating potential involvement of HSP system in CSCs. Indeed, recent findings have supported role of HSP system in functional features of CSCs 18, 19. For instance, upregulation of HSP90 was found in CSC-like cells derived from irradiated breast cancer cells 19. Positive correlation between the expression of HSP70 and stemness markers was also observed in esophageal cancer cells 19. Moreover, ablation of HSP70 delayed tumor initiation and prevented metastasis in the murine model of breast cancer via eradication of TICs/CICs 20. Hence, targeting the HSP system could be an effective strategy for eliminating both CSCs and non-CSC populations and thereby treating cancer.

Numerous anticancer drugs targeting HSP system has been developed for the treatment of cancer patients. Various HSP90 inhibitors with different structural backbones are currently under many pre-clinical and clinical investigations 21, 22. Similarly, several small-molecule inhibitors, antibodies, and peptide aptamers targeting HSP70 have shown antitumor effects in vitro and in vivo 23-27. However, none of these inhibitors have been approved for the clinical use due to their cytotoxicities, structural instability, low affinity, and/or relatively low potency 23, 28; most of these inhibitors have only been preclinically evaluated with few entered into clinical trials 23, thus emphasizing the necessity of developing novel inhibitors effectively targeting the HSP system.

Here, we demonstrate the preclinical evidence supporting the role of HSP system, especially that controlled by HSP70 via transcriptional upregulation in CSCs. Hence, we have focused on the development of HSP70 inhibitors as a therapeutic agent targeting both CSCs and non-CSC populations. To this end, we have screened a large chemical library of natural products and identified evodiamine (Evo) as a novel HSP70 inhibitor. Evo suppressed both bulk non-CSCs and rare CSCs in lung, colon, and breast cancer, exhibiting potent antitumor activities in vitro and in vivo without overt toxicity. Mechanistically, Evo disrupted the HSP system by directly interacting with the N-terminal nucleotide binding domain of HSP70. Our results show that Evo is a novel anticancer drug with a broad application targeting both non-CSCs and CSCs in various cancers.

## Methods

### Cell culture

We used human lung cancer cell lines (A549, H1299, H460, and H226B), lung cancer cell lines with acquired chemoresistance [paclitaxel-resistant H460 cells (H460/PcR), cisplatin-resistant H1299 cells (H1299/CsR), and pemetrexed-resistant H1299 cells (H1299/PmR)], the breast cancer cell line MDA-MB-231, the colorectal cancer cell line HCT116, and normal cell lines derived from mouse normal liver epithelium (NCTC1469), human lung epithelium (HBE), human colon fibroblast (CCD-18Co), human breast epithelium (MCF10A), mouse hippocampus (HT-22), and human lung fibroblast (Wi38). A549, H1299, H460, MDA-MB-231, and Wi38 cells were purchased from the American Type Culture Collection (ATCC, Manassas, VA, USA). NCTC1469 cells were purchased from Korean Cell Line Bank (KCLB, Seoul, Republic of Korea). H226B cells were kindly provided by Dr. John V. Heymach (MD Anderson Cancer Center, Houston, TX, USA). HCT116 and CCD-18Co cells were kindly provided by Dr. Sang Kook Lee (College of Pharmacy, Seoul National University, Seoul, Republic of Korea). HBE cells were kindly provided by Dr. John D. Minna (University of Texas Southwestern Medical Center, Dallas, TX, USA). HT-22 cells were provided by Dr. Dong Gyu Jo (College of Pharmacy, Sungkyunkwan University, Suwon, Republic of Korea). MCF10A cells were kindly provided by Dr. Sunghyouk Park (College of Pharmacy, Seoul National University, Seoul, Republic of Korea).

MDA-MB-231, NCTC1469, HT-22, CCD-18Co, and Wi38 cells were cultured in DMEM supplemented with 10% fetal bovine serum (FBS) and 1% antibiotics (all from Welgene, Kyeongsan-si, Republic of Korea). NSCLC cell lines, those with acquired resistance to chemotherapy, and HCT116 cells were cultured in RPMI 1640 supplemented with 10% fetal bovine serum (FBS) and 1% antibiotics (Welgene). NSCLC cell lines with acquired resistance to chemotherapy were generated by continuous exposure to corresponding anticancer drugs for more than six months. HBE cells were cultured in in K-SFM (Thermo Fisher Scientific (Waltham, MA, USA) supplemented with 5 ng/mL recombinant epidermal growth factor (EGF), 50 μg/mL bovine pituitary extracts, and antibiotics. MCF10A cells were cultured in DMEM/F12 (Welgene) supplemented with 10% FBS, 50 μg EGF, 250 μg hydrocortisone, 5 mg insulin, 50 μg cholera toxin, and 1% antibiotics.

Human cancer cell lines were authenticated and validated using AmplFLSTR identifier PCR Amplification Kit (Applied Biosystems, Foster, CA; cat. No. 4322288) in 2013, 2016, and 2020. Short tandem repeat (STR) DNA profiles for human cancer cell lines used in this study are listed in **Table S1**. Raw data of STR profiles are included in **Appendix 1** of Supplementary Materials. Cells passed for fewer than 6 months after receipt or resuscitation of validated cells were used in this study. We also routinely tested mycoplasma contamination of cultured cells and confirmed the cell lines used in this study were mycoplasma-free. A representative result of mycoplasma contamination in cancer cell lines is shown in **Figure S1**.

### Reagents

Antibodies against pAkt (S473), Akt, pSrc (Y416), Src, pMEK (S217/221), MEK, Nanog, and cleaved caspase-3 were purchased from Cell Signaling Technology (Danvers, MA, USA). Antibodies against cleaved PARP and HIF-1α and Matrigel were purchased from BD Biosciences (San Jose, CA, USA). Primary antibodies against 6x-His tag, Ubiquitin, and Actin were purchased from Santa Cruz Biotechnology (Santa Cruz, CA, USA). Antibodies against HSP70, HSP90, and Hop were purchased from Enzo Life Science (Farmingdale, NY, USA). Antibodies against Oct4 and Sox2 were purchased from Abcam (Cambridge, UK). Horseradish peroxidase (HRP)-conjugated secondary antibodies were purchased from GeneTex (Irvine, CA, USA). Ni-NTA agarose and fluorescence (Alexa Fluor 488 and Alexa Fluor 594)-conjugated secondary antibodies was purchased from Thermo Fisher Scientific. Biotinylated secondary antibodies were purchased from Bethyl Laboratories (Montgomery, TX, USA). ATP-agarose was acquired from Innova Biosciences (Cambridge, UK). Propidium iodide (PI), 3-(4,5-dimethylthiazol-2-yl)-2,5-diphenyl tetrazolium bromide (MTT), and other chemicals were purchased from Sigma-Aldrich (St. Louis, MO, USA) unless otherwise specified. The detailed information on used primary and secondary antibodies, including vendor, catalogue number, application, and dilution ratio (or concentration) is listed in **Table S2**.

### Isolation of pOct4-GFP^High^, pOct4-GFP^Low^, pNanog-GFP^High^, and pNanog-GFP^Low^ populations

H1299 and H460 cells stably transfected with GFP reporter vectors carrying human *POUSF1* or *NANOG* promoters (H1299/pOct4-GFP, H1299/pNanog-GFP, H460/pOct4-GFP, and H460/pNanog-GFP) were sorted using a FACS Aria III flow cytometer (BD Biosciences) for further in vitro experiments. When necessary, cells were treated with Evo for 2 days and then subjected to FACS. Gating strategies to determine the GFP^high^ and GFP^low^ populations, as an indicator of each promoter activity, in these cells were shown in**Figure S2**.

### Synthesis of evodiamine and biotinylated evodiamine

Chemical synthesis of evodiamine (Evo) was performed as described previously 29. The detailed procedures for synthesizing Evo and biotinylated Evo are depicted in **Figures S3-S4**. ^1^H-NMR spectra of synthesized and commercial Evo are included in **Appendix 2** of Supplementary Materials.

### MTT assay

Cells were seeded into 96-well plates at a density of 2 x 10^3^ to 1 x 10^4^ cells/well and allowed to attach for 24 h. Cells were treated with vehicle or the indicated concentrations of test compounds diluted in complete media for 2 days, after which they were treated with MTT solution (final concentration of 500 μg/mL) and incubated for 2-4 h at 37 °C. The formazan products were dissolved in DMSO, and the absorbance of each well was measured at 570 nm. The data are presented as a percentage of the control group. The half maximal inhibitory concentration (IC_50_) of Evo in each cell line is determined by nonlinear regression analysis using Graphpad Prism software (version 8, GraphPad Software, Inc., La Jolla, CA, USA). Synergistic effect of the combinatorial treatment between Evo and chemotherapeutic drugs (carboplatin and paclitaxel) were determined by calculating the combination index as described in our previous report 30. Combination index was calculated by dividing the expected growth inhibition rate (the growth inhibition rate of Evo multiplied by the growth inhibition rate of a chemotherapeutic drug) by the observed growth inhibition rate. An index more than 1 indicates synergistic effect and less than 1 indicates less than additive effect 30.

### Sphere formation assay

Cells were seeded on ultra-low attachment 96-well plates (Corning, Corning, NY, USA) in spheroid medium [DMEM-F12, supplemented with B27 supplements (Thermo Fisher Scientific, Waltham, MA, USA), 20 ng/mL EGF, 20 ng/mL bFGF, and 1% antibiotics]. Cells grown under sphere-forming conditions were treated with vehicle or the indicated compounds at varying doses. For using vehicle- or Evo-treated cells, cells were grown under sphere-forming conditions without drug treatment. Cells were incubated at 37 °C and 5% CO_2_ for 2 weeks or until spheres formed and reached above 150 µm^2^. Spheres were imaged, and the diameter of spheres and the number of spheres above 30 or 100 μm in diameter were determined using ImageJ software (National Institutes of Health, Bethesda, MA, USA).

### Aldehyde dehydrogenase (ALDH) assay

The AldeRed ALDH assay kit (Merck Millipore, Billerica, MA, USA) was used to identify the cell population with high ALDH enzymatic activity. First, 1 x 10^6^ H1299 cells were suspended in AldeRed buffer and stained with AldeRed A588 at 37 °C for 40 min. Each group contained a blank sample (AldeRed A588 alone) and a positive control sample (AldeRed A588 plus DEAB). The fluorescence intensity was obtained by flow cytometric analysis, and the sorting gates were established using a sample with DEAB treatment (negative control). The ALDH^high^ and ALDH^low^ populations were sorted using a FACS Aria III flow cytometer (BD Biosciences) for further in vitro experiments.

### Anchorage-dependent colony formation assay

Cells were seeded into 6-well plates at a density of 300 cells/well and treated for two weeks with various concentrations of Evo diluted in complete medium. The drug-containing medium was changed once or twice a week. After incubation, colonies were fixed with 100% methanol, stained with 0.02% crystal violet solution, and washed with deionized water several times. Colonies were imaged and counted using ImageJ software.

### Soft agar colony formation assay

Cells were mixed with sterile 1% agar solution (final concentration of 0.4%) and poured onto 1 % base agar in 24-well plates. Evo diluted in complete medium was added to the agar after solidification of the top agar. Cells embedded in the top agar were incubated for 2 weeks at 37 °C with 5% CO_2_, and the medium was changed twice a week. After incubation, colonies were stained with MTT solution, imaged and counted.

### Analysis of morphological changes in the cell nucleus

H1299, H460, HCT116, and MDA-MB-231 cells were treated with different doses (0, 1, and 5 μM) of Evo for 48 h. After treatment, the cells were incubated with Hoechst 33342 (Thermo Fisher Scientific) diluted in fresh serum containing media (20 μM) for 30 min, then observed under fluorescent microscope. The number of cells with condensed, fragmented, or degraded nuclei was counted.

### Annexin V-FITC/PI double staining

Cells were treated with increasing concentrations of Evo for 2 days. Adherent and floating cells were collected and washed with PBS. Cells were stained with Annexin V-FITC and PI using the commercially available Annexin V-FITC/PI double staining kit (BD Bioscience). Fluorescence intensity was analyzed by flow cytometry using a FACSCalibur^®^ flow cytometer (BD Biosciences) and analyzed using Flowing software (Cell Imaging and Cytometry (CIC) Core, Turku Bioscience) 31.

### Western blot analysis

Cells were treated with various concentrations of Evo for 2 days. Total cell lysates were prepared with modified RIPA lysis buffer (50 mM Tris-HCl [pH 7.4], 150 mM NaCl, 1 mM EDTA, 0.25% sodium deoxycholate, 1% Triton X-100) containing various protease and phosphatase inhibitors (100 mM NaF, 5 mM Na_3_VO_4_, 1 mM PMSF, 1 μg/mL aprotinin, 1 μg/mL leupeptin, and 1 μg/mL pepstatin). Equal amounts of protein (25-50 μg) were subjected to SDS-PAGE though 6-12% gels and electrophoretically transferred onto polyvinylidene difluoride (PVDF) membranes (ATTO Corp., Tokyo, Japan). Membranes were submerged into blocking buffer [5% nonfat dry milk in Tris-buffered saline (TBS) containing 0.01% Tween-20 (TBST)] for 1 h at room temperature before they were incubated overnight at 4 °C with primary antibodies diluted in 3% BSA in TBST (1: 1,000). Membranes were then washed multiple times with TBST and incubated with secondary antibodies diluted in 5% nonfat dry milk in TBST (1: 5,000) for 1 h at room temperature. Finally, the membranes were washed multiple times with TBST, and protein bands were visualized using an enhanced chemiluminescence (ECL) detection kit (Thermo Fisher Scientific).

### Real-time PCR

Total RNA was prepared using an easy-BLUE total RNA extraction kit (Intron Biotechnology, Sungnam-si, Kyunggi-do, Republic of Korea) according to the manufacturer's recommended procedure. We used a SYBR Green-based qPCR master mix solution (Enzynomics, Daejeon, Republic of Korea) and gene-specific primers. All real-time PCR assays were performed on an Applied Biosystems 7300 Real-Time PCR System (Thermo Fisher Scientific). The following thermocycler conditions for real-time PCR were applied: pre-incubation at 95 °C for 15 min; 40-70 cycles of 95 °C for 10 s, 60 °C for 15 s, and 72 °C for 30 s; and a final melt curve analysis to determine reaction specificity. Relative quantification of mRNA expression was performed using the comparative CT (cycle threshold) method as described in a previous report 32. The primer sequences used in the PCR assays are shown in **Table S3**.

### Transfection

For transient transfection, cells were transfected with expression vectors or siRNAs using the JetPrime transfection reagent (Polyplus-Transfection SA). To generate stable cell lines, H1299 cells were transduced with lentiviral particles containing control vector (shCon; pLKO.1) or HSP90 or HSP70 shRNAs (Sigma-Aldrich).

### Cloning and extraction of recombinant His-tagged HSP70 proteins

The human *HSP1A1* gene (full-length, N-terminal, and C-terminal domains) was cloned into pET28a vectors with a N-terminal (His)_6_ tag. *E. coli* BL21 cells were transformed with the construct and were cultured in lysogeny broth (LB) medium at 37 °C until the absorbance at 600 nm reached 0.5. Protein expression was induced by adding 0.2 mM isopropyl-β-*d*-thiogalactopyranoside (IPTG) and then incubating for 6 h at 37 °C. After centrifugation, cell pellets were resuspended in lysis buffer [50 mM Tris-HCl (pH 8.0), 150 mM NaCl, 1% Triton X-100, 10% glycerol, 70 μL of β-mercaptoethanol, and protease inhibitor cocktail]. After sonication and centrifugation, the supernatant was incubated with Ni-NTA agarose beads and kept at 4 °C. Mutant HSP70 carrying point mutation at amino acid residue responsible for ATP binding (R272K or R342K) was generated using QuickChange Lightning site-directed mutagenesis kit under the manufacturer's recommended procedure (Agilent Technologies, La Jolla, CA, USA).

### Immunoprecipitation and pulldown assay

For immunoprecipitation analysis, cells treated with vehicle or Evo (5 μM) for 6 h were washed with ice-cold PBS twice and then harvested by IP lysis buffer [20 mM Tris-HCl (pH 7.5), 150 mM NaCl, 0.5% NP-40, 1 mM MgCl_2_, 10% glycerol, 100 mM NaF, 5 mM Na_3_VO_4_, 1 μg/mL aprotinin, 1 μg/mL leupeptin, and 1 μg/mL pepstatin] for 10 min on ice. After centrifugation at 13,000 rpm for 10 min at 4 °C, supernatants were harvested, and protein concentration was determined by the BCA assay. 1 mg of protein was immunoprecipitated with anti-HSP70 antibodies overnight at 4 °C in lysis buffer. Protein G agarose beads were added and incubated for additional 2 h. The beads were collected by centrifugation (3,000 rpm for 2 min at 4 °C) and washed six times (three times with lysis buffer and three times with PBS). Bound proteins were extracted by boiling with 5x SDS-PAGE sample buffer for 5 min at 95 °C. Proteins were resolved by SDS-PAGE, transferred onto PVDF membranes, and then subjected to Western blot analysis as described above.

For ATP pulldown assay, recombinant proteins of HSP70 and HSP90 were incubated with or without 5 µM Evo for 4 h at 4 °C. The beads were collected and washed once with phosphate-buffered saline (PBS) containing 0.01% Tween-20 (PBST) and twice with PBS, and proteins were extracted by boiling with 5x SDS-PAGE sample buffer for 5 min at 95 °C. Proteins were analyzed as described above.

For Biotin-avidin pulldown assay, cell lysates were incubated with vehicle (DMSO) or 5 µM biotinylated-Evo for 4 h at 4 °C. After that, streptavidin-agarose were added and incubated for 2 h at 4 °C. The beads were collected and washed once with PBST and twice with PBS, and proteins were extracted by boiling with 5x SDS-PAGE sample buffer for 5 min at 95 °C. Proteins were analyzed as described above.

### Drug affinity responsive target stability (DARTS)

DARTS was performed according to the previously published procedure with some modifications 33, 34. Briefly, 35 μg of purified HSP70 proteins were treated with vehicle (DMSO) or up to 500 μM Evo (final 1% DMSO) for 30 min at 4 °C, and then incubated with proteinase K (proteinase K:protein ratio 1:100) for 15 min at room temperature. After terminating the reaction by adding 5x SDS-PAGE sample buffer and boiling for 5 min at 95 °C, lysates were resolved by 8% SDS-PAGE, transferred onto PVDF membranes, and further subjected to Western blot analysis as described above.

### Limiting dilution assay

Cells were treated with vehicle (DMSO) or Evo for three days and then harvested by trypsinization. Live cells, as confirmed using a trypan blue exclusion assay, were diluted in PBS, mixed with Matrigel (ratio 1:1), and then inoculated into the right flanks of NOD/SCID mice. The incidence of tumor formation was determined. Tumor-initiation fraction of vehicle- or Evo-treated groups was determined by using Extreme Limiting Dilution Analysis (ELDA) online software (http://bioinf.wehi.edu.au/software/elda/) 35.

### Immunofluorescence

The expression levels of cleaved caspase-3 (Cl-Cas3) and Oct4 in tissues or tumors were evaluated by immunofluorescence staining using paraffin-embedded tissues, tumors derived from the H460 xenograft model, PDX models, or lungs from *Kras^G12D/+^* transgenic mice, as previously described 36. Briefly, sections of formalin-fixed, paraffin-embedded (FFPE) tissue specimens were deparaffinized, rehydrated, and treated with citrate-based antigen unmasking solution (Vector Laboratories, Burlingame, CA, USA) for antigen retrieval. The slides were treated with 0.3% hydrogen peroxide solution and then incubated with blocking solution (5% normal serum in TBS containing 0.025% Triton X-100) for 1 h at room temperature. The slides were incubated with primary antibodies (1:100 dilution) overnight at 4 ºC. The slides were washed multiple times with wash buffer (TBS containing 0.025% Triton X-100), incubated with fluorochrome-labeled secondary antibodies (Thermo Fisher Scientific) for 1 h at room temperature, and then washed several times with wash buffer. The slides were counterstained with 4′,6-diamidino-2-phenylindole (DAPI) and observed under a fluorescence microscope (Zeiss Axio Observer Z1, Carl Zeiss AG, Oberkochen, Germany).

### Immunohistochemistry

The HSP70 expression in tumors from the H460 xenograft model and the PDX models were analyzed by immunohistochemistry (IHC). Sections of formalin-fixed and paraffin-embedded tissue specimens were deparaffinized, rehydrated, and then subjected to antigen retrieval using the citrate-based antigen unmasking solution (Vector Laboratories). After treatment with 0.3% hydrogen peroxide solution, slides were incubated with blocking buffer (5% normal serum in TBS containing 0.025% Triton X-100) for 1 h at room temperature. Slides were incubated with primary antibodies overnight at 4 ºC and then with a biotinylated secondary antibody (Bethyl laboratories) for 1 h at room temperature. Solutions A and B (ABC-Elite, Vector Laboratories) were added simultaneously for 30 min, and signals were detected using a 3,3'-diaminobenzidine (DAB) substrate kit (Vector Laboratories). Slides were further counterstained with hematoxylin.

### Animal experiments

All animal procedures were performed according to protocols approved by the Seoul National University Institutional Animal Care and Use Committee (approval No. SNU-131202-2). Mice were fed standard chow and water ad libitum and housed in a temperature- and humidity-controlled facility with a 12-h light/12-h dark cycle. For the xenograft experiment, H460 cells or patient-derived tumors were subcutaneously inoculated into the right flanks of 6-week-old NOD/SCID mice. After the tumor volume reached 50-150 mm^3^, the mice were randomly grouped, and Evo (20 mg/kg) or vehicle (10% DMSO and 40% PEG400 diluted in distilled water) were administered via oral gavage six times per week for 3 weeks. Evo was dissolved in DMSO and diluted in 40% PEG400 solution). Tumor growth was determined by measuring the short and long diameters of the tumor with a caliper, and the tumor volume was calculated using the following formula: tumor volume (mm^3^) = (small diameter)^2^ × (large diameter) × 0.5. Body weight was recorded twice per week to monitor toxicity.

In experiments using *Kras^G12D/+^* transgenic mice 37, three-month-old mice were randomized and orally treated with vehicle or Evo (20 mg/kg) for 8 weeks. Bioluminescence images were obtained using IVIS-Spectrum microCT and Living Image (ver. 4.2) software (PerkinElmer, Alameda, CA, USA) using an MMPSense 680 probe (PerkinElmer; 2 nmol/150 μl in PBS). The instrument was operated according to the manufacturer's instruction. The mice were euthanized, and tumor formation in the Evo and vehicle treatment groups was evaluated and compared. Microscopic evaluations of lung tissue after hematoxylin and eosin (H&E) staining were also performed to measure the mean tumor number (N) and volume (V) in a blinded fashion. The tumor volume was calculated using the formula described above. The tumor burden was calculated using the following formula: number of tumors × the average of tumor volume. The number and size of tumors were calculated in five sections uniformly distributed throughout each lung.

### In silico analysis

We used publicly available datasets deposited in the GEO (National Center for Biotechnology Information): GSE17537 38, GSE24747, GSE38678 39, GSE41271 40, GSE58812 41, and GSE67966 for GSEA; GSE3141 42, GSE17536 38, and GSE1456 43 for analysis of the association of the HSPA1A expression with prognosis of patients with lung, colon, and breast cancer. Raw data comprising gene expression levels and clinical information for each patient sample (such as histology, survival status, and duration of survival) were manually downloaded and analyzed. GSEA was performed using GSEA software (Broad Institute, Massachusetts Institute of Technology, Cambridge, MA, USA) with GO genesets (obtained from the Molecular Signatures Database) for heat shock protein binding or chaperone binding. The HSPA1A^High^ and HSPA1A^Low^ groups were defined based on the median value of the data in each dataset. A Kaplan-Meier survival curve was used to show differences in the survival of lung cancer patients. The log-rank test was used to determine significance.

### Molecular docking studies

AutoDock Vina software (The Scripps Research Institute, La Jolla, CA, USA) 44 was used in our docking simulation studies. The docking structure template of human HSP70 was obtained from Protein Data Bank (PDB ID**:** 2E8A). The coordinate of AMP-PNP and ATP were extracted from the Protein Data Bank (PDB ID**:** 2E8A and PDB ID**:** 3D2E, respectively). The structure of Evo compound was created using the ChemDraw program (PerkinElmer, Waltham, MA, USA). The grid maps for docking studies were centered on the nucleotide-binding sites and comprised 40 × 40 × 40 points with 1.0 Å spacing after AMP-PNP was removed from the complex structure. To validate the docking program, we implemented a control docking experiment with AMP-PNP in the HSP70 complex structure (PDB ID: 2E8A). AMP-PNP was predicted to dock into the nucleotide-binding pocket of HSP70 as in the actual complex structure with a binding energy of -9.7 kcal/mol. AutoDock Vina program was run with four-way multithreading, and the other parameters were default settings in AutoDock Vina program. Structural alignment and docking simulation result figures were created by the PyMOL Molecular Graphics System (Version 2.0, Schrödinger, LLC).

### Statistics

The data are presented as the means ± SD. All in vitro experiments were independently performed at least three times, and a representative result is presented. The data were calculated or analyzed with Microsoft Excel software (Microsoft Corp., Redmond, MA, USA) or Graphpad Prism software. Statistical significance was determined using two-tailed Student's t-test or one-way analysis of variance (ANOVA). An F-test for equality of variances was performed to ensure the same variance of two test groups. The Brown-Forsythe test for the equality of variances was performed to ensure the same variance of more than three experimental groups. The Shapiro-Wilk test were performed to determine whether the in vitro or in vivo data follows a normal distribution. *P* values of less than 0.05 was considered significant.

## Results

### Activation of the Hsp system in CSCs through transcriptional upregulation of Hsp components, especially HSP70

**Figure 1:**
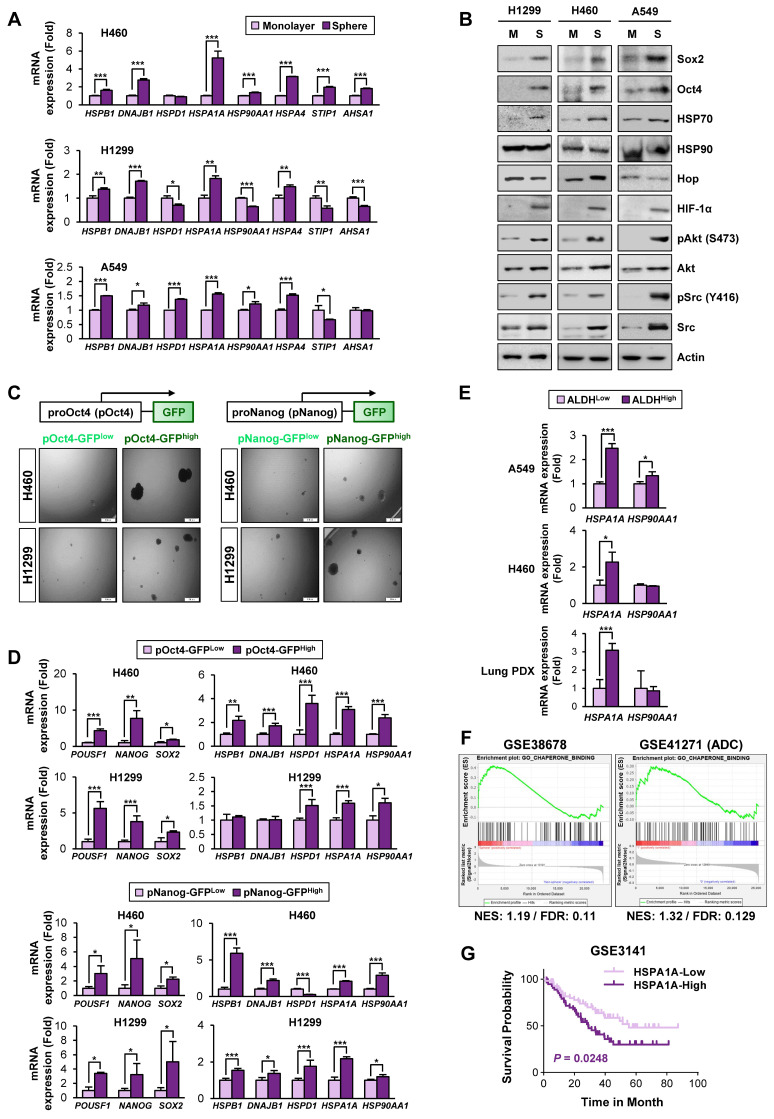
** Association of the Hsp system with the acquisition of CSC phenotypes.** (**A, B**) Expression of the components of the HSP system (mRNA: *HSPB1*, *DNAJB1*, *HSPD1*, *HSPA1A*, *HSP90AA1*, *HSPA4*, *STIP1*, *AHSA1*; protein: HSP70, HSP90, Hop) stemness-related markers (Sox2 and Oct4), HSP70, HSP90, and total or phosphorylated forms of client proteins of the HSP system (HIF-1α, Akt, and Src) in the indicated NSCLC cells grown in monolayer (M) and those grown under sphere-forming conditions (S) as evaluated by real-time PCR (*n* = 3) (**A**) and Western blot (**B**) analyses. (**C, D**) Sphere-forming capacity (*n* = 3) (**C**) and mRNA expression of CSC markers and components of the HSP system (*n* = 3) (**D**) in pOct4-GFP^Hgh^ vs. pOct4-GFP^Low^ and pNanog-GFP^High^ vs. pNanog-GFP^Low^ populations in H1299 and H460 cells. (**E**) Real-time PCR analysis of *HSPA1A* and *HSP90AA1* mRNA expression in ALDH^High^ vs. ALDH^Low^ populations in A549 and H460 NSCLC cell lines and primary cultured cells of PDX tumors (*n* = 3). (**F**) GSEA of publicly available data for the HSP system-related gene sets in NSCLC and tumors derived from patients with lung adenocarcinoma (ADC). (**G**) Kaplan-Meier survival analysis for the association of *HSPA1A* expression with overall survival of patients with lung cancer, determined by analysis of a GSE3141 dataset. All in vitro experiments were performed at least three times. The bars represent the mean ± SD; *P < 0.05, **P < 0.01, and ***P < 0.001, as determined by a two-tailed Student's *t*-test by comparison with the control (Con) group. Scale bars, 100 μm (**C**).

Based on the previous studies that suggest the impact of the HSP system on CSC behavior 13, we assessed expression profiles of the HSP system components [*HSPB1* (encodes Hsp27), *DNAJB1* (encoding Hsp40), *HSPD1* (encoding Hsp60), *HSPA1A* (encoding HSP70), and *HSP90AA1* (encoding HSP90)] in NSCLC CSCs. Given the deficit of reliable biomarkers for NSCLC CSCs, we obtained potential CSC subpopulations from NSCLC cell lines based on general traits of CSCs, including tumor sphere-forming capacities 45, expression of CSC markers, such as Oct4, Nanog, and Sox2 46, and ALDH activity 47. We found consistent upregulation of *HSPB1*,* DNAJB1*, *HSPA1A*, and* HSPA4* with a greatest increase in *HSPA1A* in the spheres derived from three different NSCLC cell lines compared with their counterparts grown in monolayer culture conditions (**Figure 1A**). The CSC traits of the spheres were validated by their capacity of propagation into second generation (**Figure S5A**) and upregulation three representative stemness markers (*POU5F1*, *NANOG*, and *SOX2*) (**Figure S5B**) compared to their corresponding parental cells grown in monolayers. Compared with those grown in monolayer culture conditions (M), the three NSCLC spheres (S) also showed increased expression of CSC marker proteins (Sox2 and Oct4), HSP70, and client proteins of the Hsp system (HIF-1α, Akt, and Src) without detectable difference in the HSP90 expression (**Figure 1B**).

Having previously shown the role of Nanog and Oct4 in the regulation of CSCs derived from lung cancer 7, we next selected putative NSCLC CSCs by establishing H460 and H1299 NSCLC cells stably transfected with green fluorescent protein (GFP) reporter vectors carrying human *NANOG* or *POUSF1* promoters (H1299/pOct4-GFP, H1299/pNanog-GFP, H460/pOct4-GFP, and H460/pNanog-GFP).

GFP^high^ vs. GFP^low^ populations were obtained by fluorescence-activated cell sorting (FACS) (**Figure S2**). Compared with their corresponding pOct4-GFP^low^ and pNanog-GFP^low^ populations, pOct4-GFP^high^ and pNanog-GFP^high^ cells exhibited significantly enhanced sphere-forming abilities 45 (**Figure 1C**). We also observed consistently increased gene expression of CSC markers (*POUSF1*, *NANOG*, and SOX2) 46, *HSPA1A*, and *HSP90AA1* in the four different pOct4-GFP^high^ or pNanog-GFP^high^ populations compared to their corresponding GFP^low^ populations (**Figure 1D**).

Prominent increases in the mRNA expression of *HSPA1A* were also observed in the sorted ALDH^high^ populations isolated from A549 and H460 cells (**Figure 1E**). The clinical relevance of these findings was validated by confirming the upregulation of *HSPA1A* mRNA expression in the sorted ALDH^high^ populations isolated from patient-derived xenograft (PDX) tumors isolated from patients with NSCLC compared with that in the corresponding ALDH^low^ populations (**Figure 1E, bottom**). Upregulation of *HSPA1A* was also found in PDX tumors of the colon and breast cancers (**Figure S6A**). Analysis of publicly available datasets using gene set enrichment analysis (GSEA) 48, 49 revealed that the Hsp-associated gene sets were significantly enriched in the putative CSC populations of lung (GSE38678), colon (GSE24747), and breast (GSE67966) cancers and in the tumors derived from patients with lung adenocarcinoma (GSE41271), colon cancer (GSE17537), and breast cancer (GSE58812) with recurrence (GSE41271 and GSE17537) or metastasis (GSE58812) [false discovery rate (FDR) < 0.25] (**Figure 1F, S6B**). We also found an association of *HSPA1A* expression with poor survival in patients with lung, colon, and breast cancers (**Figure 1G, S6C**). Hence, the HSP system is likely activated in CSCs at least in part due to the increased expression of various Hsp components, especially HSP70.

### Role of the Hsp system in CSCs

**Figure 2:**
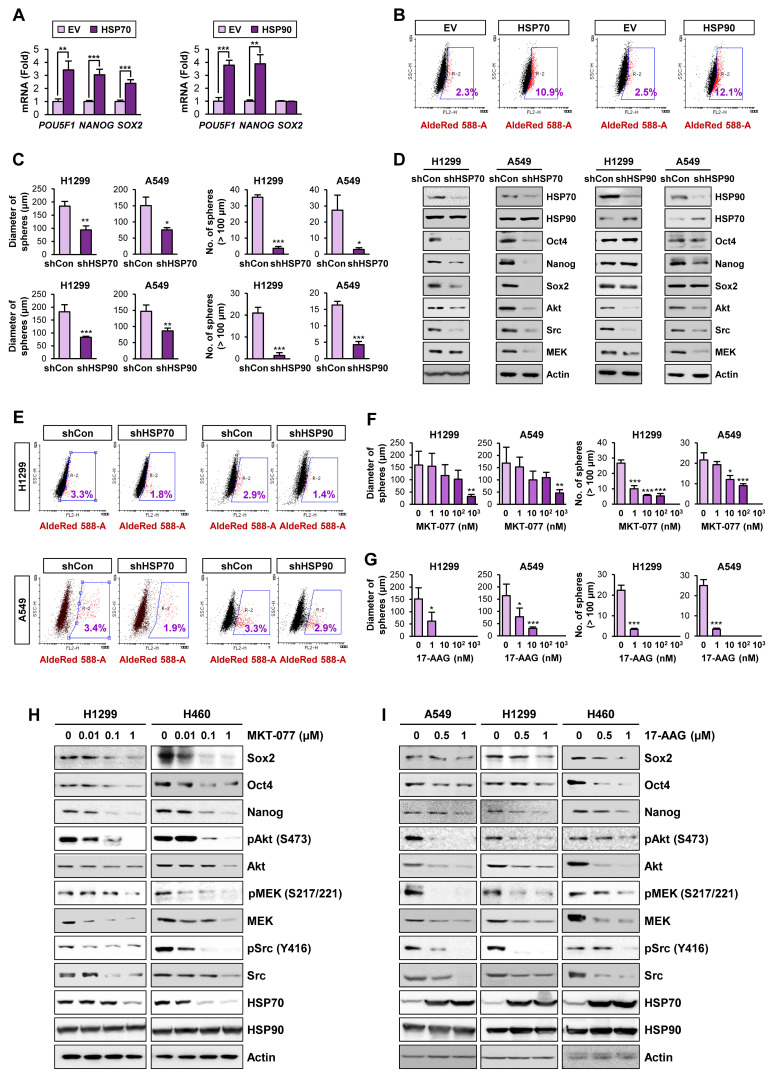
** Role of the HSP system in the acquisition of CSC phenotypes in NSCLC.** (**A, B**) Real-time PCR analysis of the indicated CSC markers (*n* = 3) (**A**) and ALDH activity (**B**) in H1299 cells carrying enforced overexpression of HSP70 or HSP90. (**C-E**) Sphere forming capacity (*n* = 5) (**C**), protein expression of the indicated CSC markers, HSP70, HSP90, and the client proteins of HSP70/HSP90 (**D**), and ALDH activity (**E**) in the indicated NSCLC cells upon shRNA-mediated ablation of HSP70 or HSP90 expression. (**F-I**) Effects of pharmacological inhibitors of HSP70 (MKT-077) and HSP90 (17-AAG) on sphere formation (*n* = 5) (**F, G**) and protein expression of CSC markers and clients of the HSP system (**H, I**) in the indicated NSCLC cells grown in normal adherent conditions as determined by the sphere formation assay (**F, G**) and Western blot analysis (**H, I**). All experiments were performed at least three times. The bars represent the mean ± SD; *P < 0.05, **P < 0.01, and ***P < 0.001, as determined by a two-tailed Student's *t*-test by comparison with the control (Con) group.

We next explored direct evidence for the functional role of the HSP system in CSCs. We observed that enforced overexpression of HSP70 or HSP90 significantly promoted the acquisition of CSC phenotypes in H1299 cells, including expression of CSC marker genes (**Figure 2A**) and ALDH activity 50 (**Figure 2B**). In contrast, H1299 and A549 cells in which HSP70 or HSP90 expression was silenced by stable transfection with specific shRNAs exhibited obvious decreases in sphere formation (**Figure 2C**) and expression of CSC marker and HSP system client proteins (**Figure 2D**) compared with their respective control cells. Notably, HSP70 silencing showed greater impact on CSC marker levels than HSP90 silencing. The ALDH^high^ population in H1299 and A549 cells was also decreased by shRNA-mediated inactivation of the HSP system (**Figure 2E**).

We next assessed the effects of pharmacological inhibitors of HSP system on CSC phenotypes. We found that treatment with HSP70 inhibitor (MKT-077) (**Figure 2F, 2H**) or HSP90 inhibitor (17-AAG) (**Figure 2G, 2I**) significantly reduced the size and the number of spheres (**Figure 2F-G**) and the expression of CSC markers and HSP70/HSP90 client proteins (**Figure 2H-I**) in the indicated NSCLC cells. Pharmacological inhibitors of HSP70 or HSP90 also showed similar inhibitory effects on sphere formation of the colon (HCT116) and breast (MDA-MB-231) cancer cells (**Figure S7A-B**) and the expression of CSC marker and HSP70/HSP90 client proteins within the spheres (**Figure S7C-D**). To confirm the specific modulation of the proteins by blockade of HSP70 or HSP90, we analyzed global protein expression patterns in cells treated with 17-AAG and MKT-077. The level of most of proteins stained with Coomassie blue was not changed by blockade of either HSP90 or HSP70, and there were certain proteins specifically modulated by treatment with each drug (**Figure S8**). Therefore, the blockade of the HSP system appears to affect a subset of specific proteins such as know Hsp90/Hsp70 client proteins.

These results suggested that the HSP system plays an important role in CSCs in lung, breast, and colon cancer. Given that HSP system is activated in various human cancers 23, these findings imply that targeting the HSP system would be a promising strategy for eliminating both CSCs and non-CSC populations thereby effectively treating various human cancers.

### Identification of evodiamine as an active compound targeting HSP system by inducing apoptosis in CSCs

Based on the role of the HSP system in stabilization of various oncoproteins in multiple types of cancer cells 35, the impact of the HSP system on CSCs, especially that controlled by HSP70 18, 19, the reported toxicities of HSP90 inhibitors 21, and cancer cell resistance to HSP90 inhibitors 51, we have attempted to develop effective inhibitors of the HSP system, especially those targeting HSP70, as a strategy to eradicate both non-CSC and CSC populations. Small molecule inhibitors have great advantages over other types of therapeutics, such as the ease of combinatorial treatment with other drugs, compatibility regardless of cell types, oral bioavailability, effective delivery to cellular targets due to high penetration yields into tissues and cells, and low manufacturing costs 52-54.

**Figure 3:**
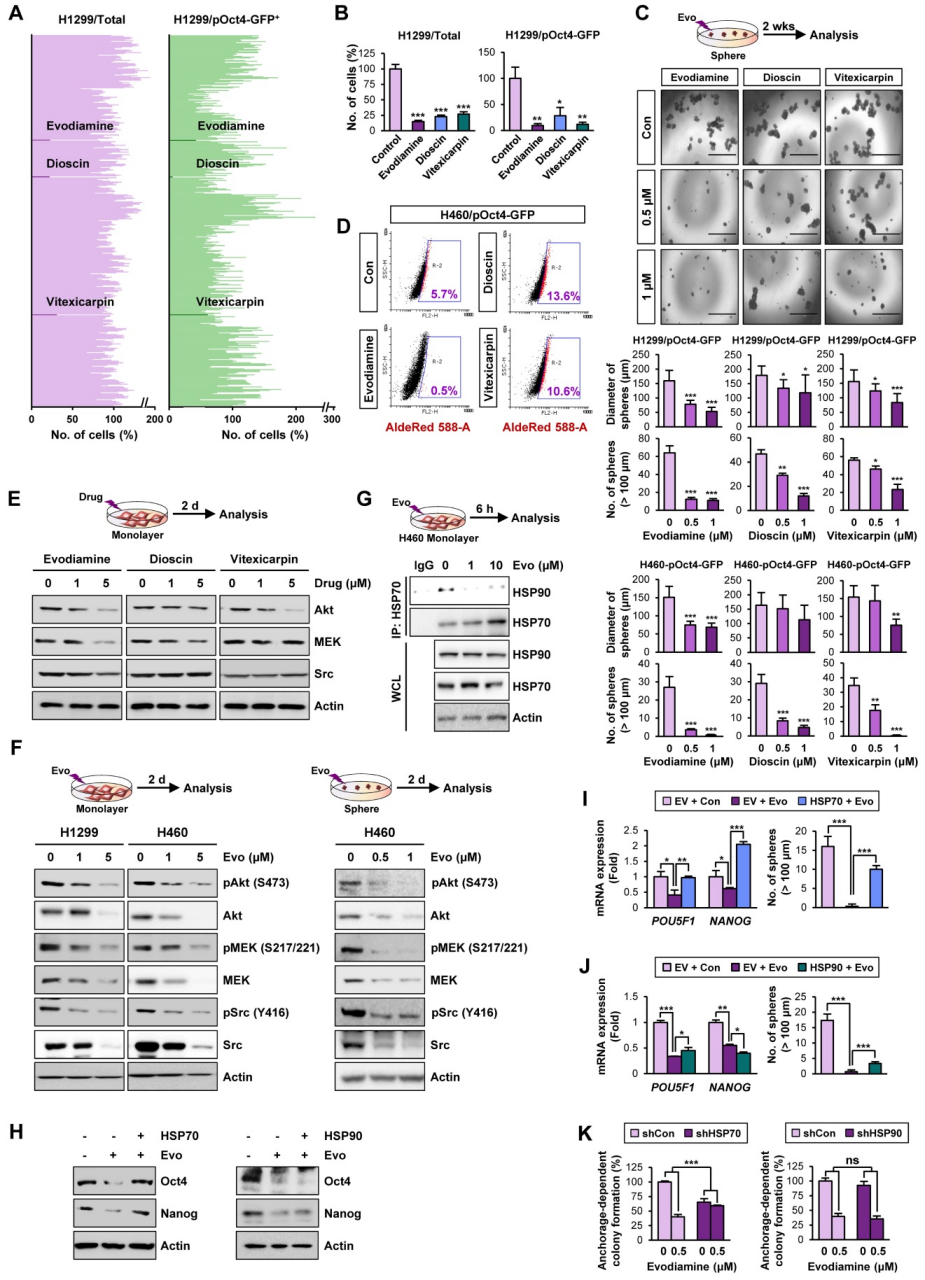
** Discovery of anti-CSC active compounds targeting the HSP system from a natural product chemical library.** (**A, B**) Screening of natural product-derived compounds utilizing H1299/total cells and H1299/pOct4-GFP^High^ subpopulations (*n* = 3) (**A**) and H460/total cells and H460/pOct-GFP^High^ subpopulations (*n* = 3) (**B**). (**C**) Sphere-forming capacity of the indicated GFP^high^ subpopulations upon treatment with Evo (evodiamine), dioscin, or vitexicarpin determined by sphere formation assay (*n* = 5). (**D**) Effect of Evo on the number of ALDH^+^ populations in H460/pOct-GFP^High^ subpopulation. (**E, F**) Western blot analysis for the effects of Evo treatment on the expression of Akt, MEK, and Src (**E, F**) and their phosphorylated forms (**F**) in H1299 and H460 cell grown in monolayer (**E, F**) or sphere-forming conditions (**F**). (**G**) Disruption of the interaction between HSP70 and HSP90 upon treatment with Evo was determined by the immunoprecipitation assay. (**H-J**) The effects of Evo treatment on protein (**H**) and mRNA expression of the indicated CSC markers (*n* = 3) (**I, J**) and sphere-forming capacity (*n* = 5) (**I, J**) in H1299 cells carrying enforced overexpression of HSP70 or HSP90 by transfection. (**K**) The effects of Evo treatment on anchorage-dependent colony formation of H1299 cells in which HSP70 or HSP90 expression was ablated by transfection with specific shRNA (*n* = 3). All experiments were performed at least three times. The bars represent the mean ± SD; *P < 0.05, **P < 0.01, and ***P < 0.001, as determined by a two-tailed Student's *t*-test by comparison with the control (Con) group. Scale bars, 1,000 μm (**C**). Evo: evodiamine; Cl-PARP: cleaved PARP; Cl-Cas3: cleaved caspase-3.

Therefore, to discover hits/candidates that could eradicate both CSCs and non-CSC populations by targeting the HSP system, we screened a large natural product chemical library consisting of 432 compounds with various chemical classes (**Table S4**) utilizing H1299 cells containing both CSC and non-CSC populations (H1299/total) and their Oct4^+^ subpopulations containing putative CSCs (H1299/pOct4-GFP). We identified three potent compounds (alkaloid, evodiamine; steroidal saponin, dioscin; flavonoid, vitexicarpin) that significantly inhibited numbers of H1299/total cells and H1299/pOct4-GFP subpopulations compared with those of untreated control cells (**Figure 3A**). Similar reduction in the numbers of H460/total cells and H460/pOct4-GFP subpopulations was also observed upon the treatment with the three compounds (**Figure 3B**). These three compounds significantly suppressed the sphere-forming capacity of H1299/pOct4-GFP and H460/pOct4-GFP cells in a dose-dependent manner with the greatest effects by evodiamine (Evo) treatment (**Figure 3C**). Treatment with Evo also revealed greatest decreases in the number of ALDH^+^ populations (**Figure 3D**) and the expression of HSP system client proteins, including Akt, MEK, and Src (**Figure 3E**). These findings along with the advantage that the synthetic method for Evo is available 29, 55 led us to choose Evo as a CSC-targeting active principle that suppresses the HSP system.

We then performed a large-scale chemical synthesis of Evo following the previously published protocol 29 (**Figure S3-4**). We confirmed the inhibitory effect of Evo on the expression of Akt, MEK, and Src and their active (phosphorylated) forms in both CSC and non-CSC populations in NSCLC (H1299, H460), colon (HCT116), and breast (MDA-MB-231) cancer cells (**Figure 3F, S9**). Moreover, the interaction between HSP70 and HSP90 proteins for their chaperone function 56 was also diminished by the Evo treatment (**Figure 3G**).

We then investigated whether HSP70 is the target for Evo. We observed that enforced overexpression (**Figure S10**) of HSP70, not HSP90, markedly restored CSC phenotypes, including expression of Oct4 and Nanog protein (**Figure 3H**) and mRNA (**Figure 3I-J**) expression and sphere-forming efficacy (**Figure 3I-J**), in Evo-treated H1299 cells. We further observed that the inhibitory effects Evo on anchorage-dependent colony formation were attenuated in H1299 cells in which HSP70 expression was stably knocked down by the use of specific shRNA (**Figure 3K**). There was no significant modulation in the inhibitory effects of Evo in H1299 cells stably knocked down HSP90 expression (**Figure 3K**). These results collectively suggest that HSP70 is as a cellular target for Evo.

### Evo displays potent anti-CSC activities in vitro and in vivo by inducing apoptosis

**Figure 4:**
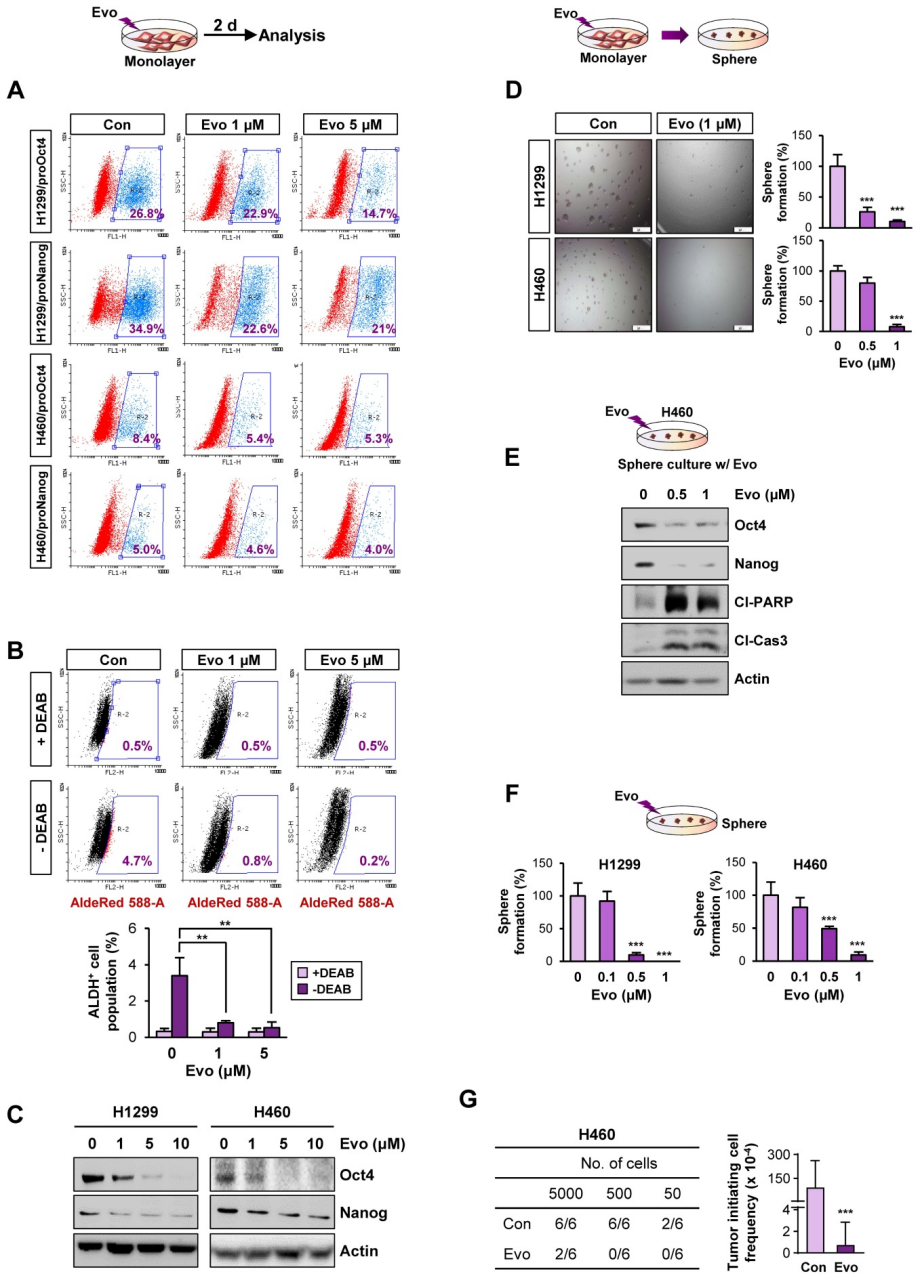
** Effects of Evo on functional features of CSCs.** (**A**) Effects of Evo on the levels of the indicated pOct4-GFP^High^ and pNanog-GFP^High^ populations of H1299 and H460 cells. (**B-E**) Effects of Evo on ALDH activity (*n* = 3) (**B**), expression of stemness markers (*n* = 3) (**C**), and sphere-forming capacity (*n* = 5) (**D**) in the indicated NSCLC cells cultured under monolayer culture conditions in the presence of Evo. (**E, F**) Effects of Evo on protein expression of stemness markers, including Oct4 and Nanog, and apoptotic markers, including cleaved PARP (Cl-PARP) and cleaved caspase3 (Cl-Cas3) (**E**) and sphere formation (*n* = 5) (**F**) of the indicated NSCLC cells cultured under sphere-forming conditions. (**G**) The effect of Evo on the tumorigenicity of H460 cells was determined by limiting dilution assay. Tumor initiating cell frequency was determined by ELDA. All in vitro experiments were performed at least three times. The bars represent the mean ± SD; *P < 0.05, **P < 0.01, and ***P < 0.001, as determined by a two-tailed Student's *t*-test by comparison with the control (Con) group (**D, F**) and one-way ANOVA with Dunnett's post-hoc test (**B**). Scale bars, 500 μm (**C**). Evo: evodiamine; Cl-PARP: cleaved PARP; Cl-Cas3: cleaved caspase-3.

We next assessed the effects of Evo on the functional features of CSCs. We found that treatment with Evo significantly decreased the number of H1299/pOct4-GFP, H1299/pNanog-GFP, H460/pOct4-GFP, and H460/pNanog-GFP cells in a concentration-dependent manner (**Figure 4A**). We observed that the ALDH^high^ cell population in H1299 cells was also markedly decreased upon treatment with Evo (**Figure 4B**). Moreover, Evo effectively suppressed Oct4 and Nanog expression (**Figure 4C**) and sphere-forming capacity (**Figure 4D**) compared to those in the vehicle-treated cells. Along with marked decreases in the expression of Oct4 and Nanog, the sphere-forming populations of H460 underwent apoptosis upon treatment with Evo, as evidenced by the increased cleavage of poly-(ADP-ribose) polymerase (PARP) and caspase-3 (**Figure 4E**). We also observed that Evo treatment reduced the growth of these spheres in a dose-dependent manner; the spheres were almost completely diminished by Evo at concentrations less than 1 μM (**Figure 4F**). Limiting dilution assays further showed that Evo-treated cells displayed markedly reduced tumorigenic activities in the recipient mice compared with those of tumor cells derived from vehicle-treated mice (**Figure 4G, Table S5**). Similar inhibitory effects of Evo on the expression of Oct4 and Nanog and sphere-forming capacity along with cleavages of PARP and caspase-3 were observed in HCT116 and MDA-MB-231 cells cultured in monolayer or sphere-forming conditions in the presence of Evo (**Figure S11A-D**). Limiting dilution assays further confirmed reduced tumorigenic activities of Evo-treated MDA231 cells (**Figure S11E, Table S5**). Together, these results suggest the anti-CSC activities of Evo.

### Evo displays potent antiproliferative activities in several types of human cancer cells by inducing apoptosis

**Figure 5:**
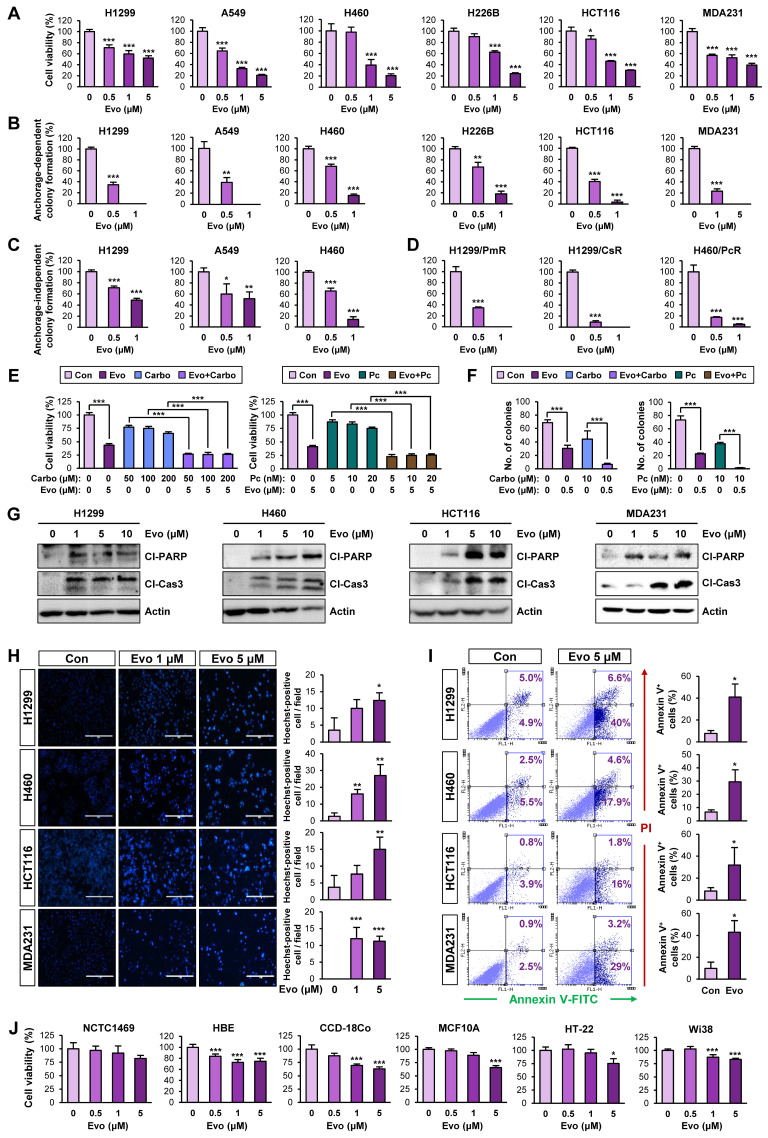
** Effects of Evo on viability, colony formation, and apoptotic activities in various types of cancer cells with minimal toxicity to normal cells.** (**A**) Effects of Evo on the viability of the indicated NSCLC, colon, and breast cancer cells were evaluated using the MTT assay (*n* = 6). (**B-D**) Anchorage-dependent (*n* = 3) (**B, D**) and anchorage-independent (*n* = 3) (**C**) colony formation of the indicated NSCLC, breast cancer, colorectal cancer cells, and NSCLC cells that are resistant to pemetrexed (H1299/PmR), cisplatin (H1299/CsR), or paclitaxel (H460/PcR) were analyzed after treatment with Evo. (**E, F**) The MTT (*n* = 6) (**E**) and anchorage-dependent colony formation (*n* = 3) (**F**) assays showing regulation of the viability (**E**) and colony-forming capacity (**F**) of the indicated NSCLC cells treated with Evo and chemotherapeutic drugs (carboplatin or paclitaxel), either alone or combination. (**G-I**) Evo-induced apoptosis was determined by Western blot analysis (**G**), Hoechst 33342 staining (*n* = 3) (**H**), and Annexin V/PI double staining (*n* = 3) (**I**). (**J**) Effects of Evo on the viability of the indicated normal cells were evaluated by the MTT assay (*n* = 6). All experiments were performed at least three times. The bars represent the mean ± SD; *P < 0.05, **P < 0.01, and ***P < 0.001, as determined by a two-tailed Student's *t*-test by comparison with the control (Con) group (**A-D, H-J**) or one-way ANOVA with Dunnett's post-hoc test (**E**). Scale bars, 400 μm (**H**). Evo: evodiamine; MDA231: MDA-MB-231; Cl-PARP: cleaved PARP; Cl-Cas3: cleaved caspase-3.

We next evaluated the effects of Evo on bulk non-CSC populations in various types of cancer cells. Evo significantly inhibited the viability (**Figure 5A, Table S6**) and anchorage-dependent colony formation (**Figure 5B**) of several cancer cell lines derived from lung, colon, and breast cancer in a concentration-dependent manner. We further confirmed the inhibitory effects of Evo on anchorage-independent colony formation of lung cancer cells (**Figure 5C**). CSCs have been proposed to induce chemoresistance 57.

Hence, we tested the effects of Evo on H1299 and H460 NSCLC cells that showed resistance to pemetrexed (H1299/PmR), cisplatin (H1299/CsR), or paclitaxel (H460/PcR) 58. We found that Evo significantly suppressed the colony-forming potential of various chemoresistant sublines (H1299/PmR, H1299/CsR, and H460/PcR) (**Figure 5D**). We next examined whether Evo could enhance the anticancer effect of conventional chemotherapeutic agents. The MTT and anchorage-dependent colony formation assays revealed that treatment with Evo significantly enhanced the inhibitory effects of cisplatin and paclitaxel on the viability (**Figure 5E, Table S7**) and colony-forming capacity (**Figure 5F, Table S7**) of NSCLC cells. These findings suggested that Evo can be applied for anti-cancer therapies in combination with standard of care, such as chemotherapy.

Consistent with the proapoptotic effect of Evo in lung cancer CSCs, treatment with Evo induced apoptosis in NSCLC cells in a dose-dependent manner, as indicated by increases in PARP and caspase-3 cleavage (**Figure 5G**), chromatin condensation (**Figure 5H**), and the annexin V-positive cell population (**Figure 5I**). Importantly, treatment with Evo (up to 5 μM) did not significantly affect the viability of normal cell lines derived from liver epithelium (NCTC1489), lung epithelium (HBE), colon fibroblast (CCD-18Co), breast epithelium (MCF10A), mouse hippocampus (HT-22), and lung fibroblast (Wi38), indicating minimal toxicity of Evo in vitro (**Figure 5J**). Altogether, these results indicate that Evo inhibits both CSC and non-CSC populations in lung, colon, and breast cancers by inducing apoptosis and exerts minimal toxicity in various normal cells.

### Antitumor effect of Evo with minimal toxicity in vivo

**Figure 6:**
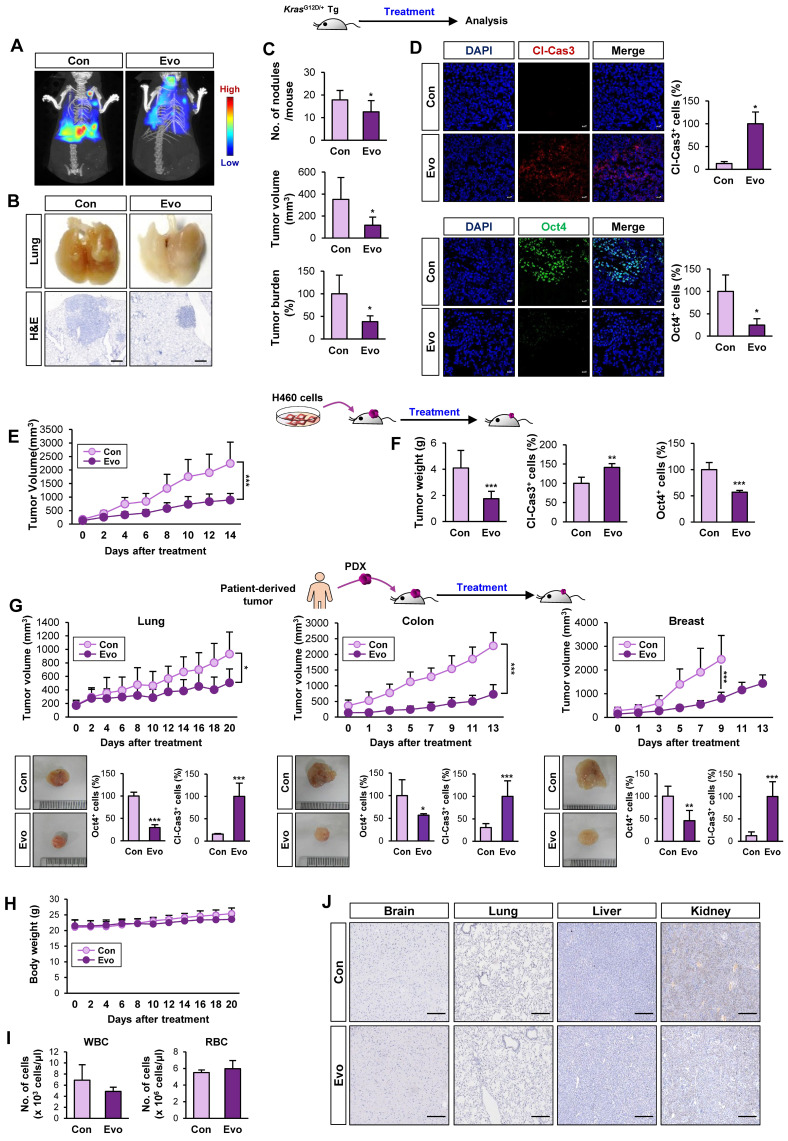
** Antitumor effects of Evo.** Inhibitory effect of Evo on the growth of lung tumors. Three-month-old *Kras*^G12D/+^ transgenic mice (*n* = 6) (**A-D**) and NOD/SCID mice carrying xenograft tumors of H460 cells (**E, F**) or lung, colon, and breast PDXs (**G, H**) were treated with vehicle or Evo (20 mg/kg) for 2 months (H460 xenograft: Con *n* = 9, Evo *n* = 18; lung PDX: Con *n* = 10, Evo *n* = 10; colon PDX: Con *n* = 6, Evo *n* = 9; breast PDX: Con *n* = 7, Evo *n* = 8). (**A, B**) Representative images of bioluminescence imaging (**A**), gross observation (**B, top**), and microscopic observation of the H&E-stained lung tissues from Kras^G12D/+^ mice (**B, bottom**). Additional bioluminescence images are shown in **Figure S12**. (**C, E, F**) Quantification of tumor multiplicity, volume, and burden in the lungs from vehicle- or Evo-treated *Kras*^G12D/+^ mice (**C**) and xenograft tumor volume (**E**) and weight (**F**) of vehicle- or Evo-treated NOD/SCID mice. (**D, G**) Immunofluorescence (IF) analysis of cleaved caspase-3 (Cl-Cas3) and Oct4 expression in the tumors from vehicle-treated control (Con) and Evo-treated mice. Representative IF images are shown in **Figure S13**. (**H-J**) Minimal toxicity of Evo in vivo. No overt differences in body weight (**H**), the WBC and RBC counts (**I**), and histological features in tissues from brain, lung, liver, and kidney (**J**) were observed between vehicle- and Evo-treated mice in the H460 tumor xenograft model. The bars represent the mean ± SD; *P < 0.05, **P < 0.01, and ***P < 0.001, as determined by a two-tailed Student's t-test by comparison with the control (Con) group. Scale bars, 500 μm (**B**), 20 μm (**D**), and 250 μm (**J**). Evo: evodiamine.

We evaluated the effects of Evo on *Kras^G12D/+^*-driven spontaneous lung tumorigenesis 37 using established transgenic mice systemically expressing *Kras*^G12D/+^ 37. We chose 20 mg/kg of Evo for in vivo experiments based on the previous literatures that showed suppression of tumor growth and inflammation by the dose 59-62. Bioluminescence imaging (**Figure 6A, S12**) and gross observation (**Figure 6B**) revealed a significant decrease in tumor formation in the lungs of Evo-treated mice after eight weeks of Evo administration. Microscopic evaluation of hematoxylin and eosin (H&E)-stained lung sections confirmed that Evo significantly suppressed tumor multiplicity, volume, and burden in the lungs of mice (**Figure 6B-C**). Immunofluorescence staining further revealed upregulation of cleaved caspase-3 (Cl-Cas3) and downregulation of Oct4 expression levels in lung tumors from Evo-treated mice compared with those in lung tumors from vehicle-treated mice (**Figure 6D**). Consistent with these findings, treatment with Evo significantly reduced the growth of H460 xenografts (**Figure 6E**). Immunofluorescence analyses revealed significant caspase-3 cleavage and marked downregulation of Oct4 levels in tumors derived from Evo-treated mice (**Figure 6F**), indicating the induction of apoptosis and inhibition of CSCs in tumors from Evo-treated mice. Administration of Evo also significantly inhibited the growth of all three PDX tumors (**Figure 6G**) along with significant increase in the cleavage of caspase-3 and marked downregulation of Oct4 levels in the tumors (**Figure 6G, S13**). During Evo treatment, we observed minimal and insignificant changes in body weight between vehicle- and Evo-treated mice (**Figure 6H**). Moreover, administration of Evo caused negligible changes in the red blood cell (RBC) and white blood cell (WBC) counts in mice (**Figure 6I**), indicating minimal hematological toxicity of Evo in vivo 63. Moreover, no detectable histological changes were observed in the major organs, such as brain, lung, liver, and kidney, from Evo-treated mice compared with those in vehicle-treated mice (**Figure 6J**). These results indicate that Evo is capable to suppress *Kras^G12D/+^*-driven lung tumorigenesis and the growth of cell line- and patient-derived tumor xenografts and exerts minimal toxicity.

### HSP70 as a cellular target of Evo

**Figure 7:**
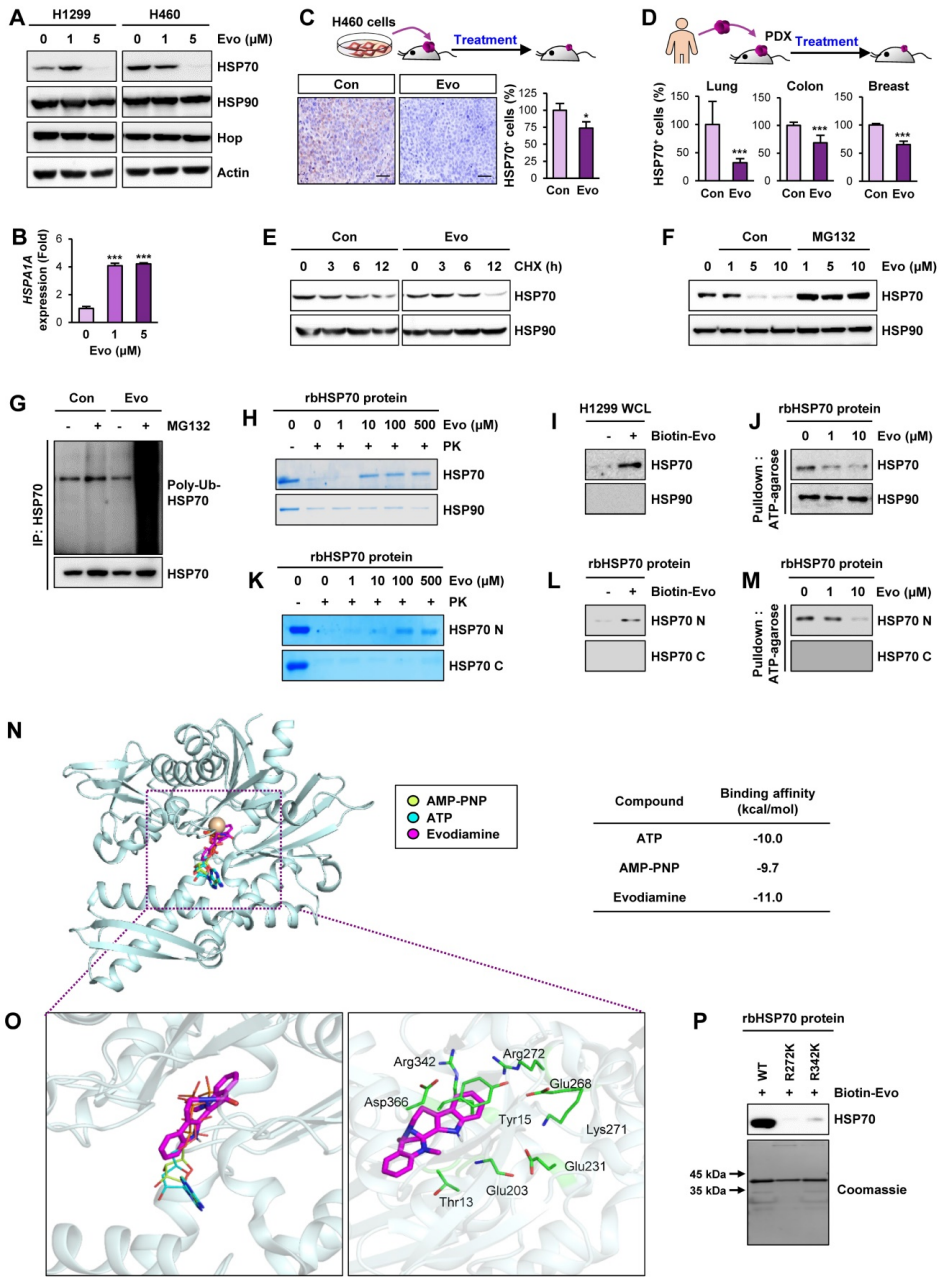
** Evo binding to NBD of HSP70 induces ubiquitin-mediated degradation of HSP70 and interruption of the HSP system.** (**A, B**) Western blot (**A**) and real-time PCR (*n* = 3) (**B**) analyses for the effects of Evo on HSP70 (**A, B**) and HSP90 (**A**) protein and/or mRNA expression in the indicated NSCLC cells grown in monolayer. (**C, D**) Immunohistochemistry (IHC) analysis of HSP70 protein levels in H460 NSCLC xenograft tumors (**C**) and PDX tumors of lung, colon, or breast cancer (**D**) from mice treated with vehicle (Con) or Evo (H460 xenograft: Con *n* = 9, Evo *n* = 18; lung PDX: Con *n* = 10, Evo *n* = 10; colon PDX: Con *n* = 6, Evo *n* = 9; breast PDX: Con *n* = 7, Evo *n* = 8). Representative IHC images of tumors from three PDX models are shown in Figure S15. (**E**) Western blot analysis for the effects of Evo on the half-life of HSP70 protein. Vehicle- or Evo-treated lung cancer cells for 2 days were exposed to CHX for up to 12 h. The level of HSP70 protein at each time point of CHX treatment was determined by Western blot analysis. (**F**) Vehicle- or Evo-treated cells were treated with MG132 (10 μM) for 6 h. HSP70 protein expression was determined by Western blot analysis (**F**). (**G**) The level of polyubiquitinated HSP70 was determined by immunoprecipitation with the anti-HSP70 antibody, followed by Western blot analysis with anti-Ubiquitin antibody. (**H-M**) Evo binding to the NBD of HSP70. (**H, K**) The binding of Evo to HSP70 in recombinant HSP70 (rbHSP70) (**H**) and the NBD domain of recombinant HSP70 (rbHSP70) (**K**) was determined by DARTS. (**I, L**) The binding of biotinylated Evo (Biotin-Evo) to HSP70 in H1299 whole cell lysates (WCL) (**I**) and the NBD domain of rbHSP70 (**L**) was determined the pulldown assay. (**J, M**) The effects of Evo on the binding of ATP to HSP70 in rbHSP70 (**J**) and the NBD of rbHSP70 (**M**) were determined by using ATP-agarose. (**N, O**) Docked position of Evo with HSP70. Binding mode of AMP-PNP, ATP, and Evo in the nucleotide-binding region of HSP70. HSP70 protein structure is shown as a cartoon model in light blue. Mg^2+^ ion was adapted from the crystal structure of HSP70 in complex with AMP-PNP (PDB ID: 2E8A) coloured in brown. AMP-PNP, ATP, and Evo ligands are represented as stick models in yellow green, cyan, and magenta, respectively (**N**). Close-view of the nucleotide-binding sites of HSP70 is represented as a green stick with carbon, nitrogen, and oxygen colored in green, blue, and red, respectively (**O**). (**P**) The binding of biotinylated Evo to rbHSP70 protein with mutations at the predicted amino acid residues for ATP binding. All experiments were performed at least three times. The bars represent the mean ± SD; ***P < 0.001, as determined by a two-tailed Student's t-test by comparison with the control (Con) group. Scale bars, 500 μm (**C**), Evo: evodiamine.

We investigated the mechanism underlying the Evo-mediated regulation of HSP system. We examined the effect of Evo on HSP70 and HSP90 expression. Intriguingly, HSP70 protein expression in H1299 and H460 NSCLC cells was suppressed by treatment with Evo in a dose-dependent manner while HSP90 expression was negligibly changed by the Evo treatment (**Figure 7A**). We further confirmed decreases in HSP70, but not HSP90, expression in H460, HCT116, MDA-MB-231 cultured in sphere-forming condition (**Figure S14**). Notably, *HSPA1A* gene expression in the Evo-treated NSCLC cells showed a marked increase compared to that in contorl cells (**Figure 7B**), suggesting a compensatory induction of the gene upon the Evo treatment-induced suppression of HSP70 protein levels. We also confirmed significantly decreased HSP70 expression in H460 NSCLC xenograft and PDX tumors from Evo-treated mice compared with those from vehicle-treated mice (**Figure 7C-D, S15**). We then investigated the effect of Evo treatment on the half-life of HSP70 protien. HSP70 protein was more rapidly degraded in Evo-treated cells than in vehicle-treated cells (**Figure 7E**).

Based on the role of ubiquitination in the regulation of protein stability 64, we investigated the role of the ubiquitin-proteasome system in the Evo-mediated downregulation of the HSP70 protein level. We observed that the Evo-mediated decreases in HSP70 protein expression were markedly restored in the presence of the proteasome inhibitor MG132 (**Figure 7F**). Moreover, the polyubiquitination of the HSP70 protein was evident in Evo-treated H1299 NSCLC cells in which proteasome machinery was inactivated by MG132 (**Figure 7G**). These findings suggest that Evo deregulates HSP70 protein expression by promoting ubiquitin-mediated proteasomal degradation.

To determine whether HSP70 as a cellular target for the activity of Evo, we performed a series of experiments. The drug affinity responsive target stability (DARTS) assay (**Figure 7H**), an analysis based on the characteristics of the reduced susceptibility of the drug-bound protein to proteolysis 33, and pulldown assay using biotinylated Evo (**Figure 7I**) showed Evo association with HSP70, but not HSP90. Structural analysis of HSP70 revealed an N-terminal nucleotide binding domain (NBD) and a C-terminal substrate-binding domain (SBD) with a flexible linker connecting the two domains 56. We observed that binding of ATP agarose with recombinant HSP70 protein was markedly blocked by the presence of Evo (**Figure 7J**). We further determined whether Evo directly binds to HSP70, and, if so, which domain of HSP70 interacts with Evo. To this end, recombinant HSP70 proteins containing the truncated N-terminal or C-terminal (C) domains were prepared. Application of the recombinant proteins to the DARTS assay (**Figure 7K**), biotinylated Evo-based pulldown assay (**Figure 7L**), and ATP agarose-based binding analysis (**Figure 7M**) consistently showed direct binding of Evo to the HSP70, especially its NBD. These results indicated HSP70 NBD as a cellular target for anti-CSC effect of Evo.

We then performed an in silico docking study to determine the binding mode of Evo to HSP70. The control docking analysis with ATP in the HSP70 complex structure (PDB ID: 3D2E) showed that ATP berthed into the NBD of HSP70 in a close proximity to the docking by AMP-PNP, the nonhydrolyzable analog of ATP 65 (**Figure 7N**). Evo docked into the NBD of HSP70 in presentation of Mg^2+^ with a stabilization energy of -11.0 kcal/mol, which is greater than that of ATP and AMP-PNP (**Figure 7N, S16**). Evo was likely to be surrounded by the well-known nucleotide-binding residues, including Thr13, Tyr15, Glu203, Glu231, Glu268, Lys271, Arg272, Arg342, and Asp366 (**Figure 7O**) 66. Among them, K271, R272 and R342 were suggested to play a central role in mediating the communication between the nucleotide-binding site and the other parts of the HSP70 ATPase domain 67. Hence, we assessed the specific binding of Evo to the ATP binding sites of HSP70 by performing a point mutation analysis. To this end, we generated bacterial HSP70 recombinant proteins carrying point mutation at R272K or R342K). A pulldown assay using biotinylated Evo revealed that the direct binding of HSP70 with Evo was almost completely diminished when the mutation was established in the nucleotide-binding residues (**Figure 7P**). These results collectively indicated that Evo disrupts the HSP system by directly binding to the NBD of HSP70 and causing its destabilization.

## Discussion

In this study, we show elevated activation of the HSP system in CSCs, possibly through transcriptional upregulation of the components of the HSP system, especially HSP70. We further show the capacity of Evo, an alkaloid derived from *Evodia rutaecarpa* 68, as an HSP70 inhibitor to suppress CSC populations of lung, breast, and colon cancer cells at submicromolar concentrations and bulk non-CSC populations at micromolar concentrations. Mechanistically, Evo directly interacts with the NBD of HSP70, thereby disrupting the protein stability of HSP70. These results suggest that targeting HSP70 would be an effective anticancer strategy to eradicate CSCs and support the use of Evo as a lead candidate for developing HSP70-targeted anticancer drugs.

Previous reports have shown the critical role of the HSP system in maintaining the survival of CSCs under various environmental stresses by mediating proper protein folding 17, 69. Members of the Hsp system play an critical role in stabilization of several client oncoproteins and have been implicated in tumor progression 12, 70, 71. Among the family, the HSP90 and HSP70 are the most studied families of HSPs 11. HSP90 constitutes multichaperone complexes with other chaperone proteins, including HSP70 12. HSP90 and HSP70 are frequently deregulated in a variety of tumor types 12, contributing to a poor prognosis and resistance to anticancer therapies 10, 13, 72. These findings imply that targeting the HSP system would be a promising strategy for eliminating both CSCs and non-CSC populations thereby effectively treating cancers, including NSCLC. Our data from potential CSCs confirmed upregulation of *HSPA1A* compared to that in bulk non-CSC subpopulations. Consistently, GSEA showed the enrichment of gene sets relating to HSP function in CSCs from patients with lung, breast, and colon cancer with recurrence or metastasis, a feature closely associated with CSC 4. In line with previous findings demonstrating upregulation of some HSP system components in murine and human embryonic SCs 73, 74, our data imply that transcriptional upregulation of HSP70 plays a role in activating the Hsp system in CSCs and promoting their self-renewal and survival. Therefore, developing treatment approaches that target HSP70 may offer a novel opportunity for eliminating both non-CSCs and CSCs in tumors.

We have therefore endeavored to develop potent, but safe, anticancer interventions targeting the HSP system. Numerous HSP90 inhibitors with various structural backbones have been developed for the treatment of cancer patients and currently under many pre-clinical and clinical investigations 21. However, none of these inhibitors have been approved for the clinical use due to their cytotoxicities, structural instability, low affinity, and/or relatively low potency 21. Hence, we focus on the development of HSP70 inhibitors as a therapeutic agent targeting both CSCs and non-CSC populations. Although HSP70-specific treatments could be a valuable and alternative strategy that targets both CSCs and non-CSCs, the toxicity has been reported in clinical trials for HSP70 inhibitors 23. Hence, it is important to develop safe yet efficacious HSP70 inhibitors. Our results show that Evo offers reasonable efficacy and an acceptable safety profile in inactivating HSP70. Previous reports have demonstrated the antitumor effects of Evo in various types of cancer cells via induction of cell cycle arrest at G2/M phase and apoptosis and inhibition of multiple signaling pathways involved in cell survival, migration, and multidrug resistance 75. The anti-CSC effects of Evo in human gastric cancer cells have been reported 76, 77; however, the cellular target by which Evo exerts its anti-CSC effects has been poorly defined, and the evaluation of the antitumor effect of Evo with clinically relevant models has rarely been performed. We show that Evo offers reasonable antitumor efficacy in clinically relevant models. Of note, despite the remarkable antitumor and anti-CSC effects of Evo, Evo showed no overt toxicities in vitro and in vivo. Furthermore, we defined HSP70 as a cellular target for antitumor and anti-CSC effects of Evo. The mechanisms by which Evo inhibits HSP70 appear to be unique and distinct from those of most currently available HSP70 inhibitors. By biochemical and in silico studies, we found that Evo was likely to disrupt the conformation of HSP70 by interacting with ATP-binding pocket of HSP70 located in the N-terminal domain 56, thereby leading to decreases in the stability of HSP70 and induction of its degradation through the ubiquitin-proteasome system. These findings suggest that Evo can be regarded as a novel hit compound or candidate thus providing a new structural backbone for the development of anticancer HSP70 inhibitors.

In summary, we show that the HSP system is activated in CSCs via transcriptional upregulation of HSP components, especially HSP70. Our findings further provide evidence that Evo effectively inactivates the HSP system in both non-CSC and CSC populations by binding to the NBD of HSP70 and inducing its degradation, eventually leading to potent antitumor activities in clinically relevant models with minimal toxicity. Further studies to evaluate the potential clinical utility of Evo in additional preclinical and clinical settings and the development of Evo derivatives with improved efficacy and/or safety profiles are warranted.

## Supplementary Material

Supplementary figures and tables, appendices.

## Abbreviations

ALDH: aldehyde dehydrogenase
CIC: cancer initiating cells
CSC: cancer stem cells
CHX: cycloheximide
DARTS: drug affinity responsive target stability
ELDA: extreme limiting dilution analysis
Evo: evodiamine
FDR: false discovery rate
HSP: heat shock protein
NBD: nucleotide binding domain
PARP: poly-(ADP-ribose) polymerase
PDX: patient-derived tumor xenograft
SBD: substrate-binding domain
SC: stem cell
TIC: tumor-initiating cells
