# Supplementary material for: Evodiamine inhibits both stem cell and non-stem-cell populations in human cancer cells by targeting heat shock protein 70
Source: Theranostics. 2021 Jan 1;11(6):2932–52. doi: 10.7150/thno.49876 (PMC7806467; doi:10.7150/thno.49876)

Supplementary materials

Evodiamine inhibits both stem cell and non-stem-cell populations in human cancer cells by targeting heat shock protein 70

Seung Yeob Hyun, Huong Thuy Le, Hye-Young Min, Honglan Pei, Yijae Lim, Injae Song, Yen T. K. Nguyen, Suckchang Hong, Byung Woo Han, Ho-Young Lee

Table S1. Short tandem repeat (STR) DNA profiles for human cancer cell lines used in this study.

Marker	H1299	H460	A549	HCT116	MDA-MB-231 (MDA231)
Amelogenin	XX	XY	XY	XX	XX
D8S1179	10, 13	12	13, 14	10, 14, 15	13
D21S11	32.2	30	29	29, 30	30, 33.2
D7S820	10	9, 12	8, 11	11, 12	8
CSF1PO	12	11, 12	10, 12	7, 10	12, 13
D3S1358	17	15, 18	16	12, 16, 17	16
TH01	6, 9.3	9.3	8, 9.3	8, 9	7, 9.3
D13S317	12	13	11	10, 12	13
D16S539	12, 13	9	11, 12	11, 13	12
D2S1338	23, 24	17, 25	24	16	21
D19S433	14	14	13	11, 12	11, 14
vWA	16, 18	17	14	17, 22	15
TPOX	8	8	8, 11	8, 9	8, 9
D18S51	16	13, 15	14, 17	15, 17	11, 16
D5S818	11	9, 10	11	10, 11	12
FGA	20	21, 23	23	18, 23	22, 23

Table S2. Antibodies used in this study.

Target	Vendor	Catalogue Number	Clone	Dilution ratio	Application1)
HSP70	Enzo	ADI-SPA-810-F	C92F3A-5	1:1000 (WB)<br>1:50 (IHC)<br>1:50 (IF)<br>1 :1000 (IP)	WB, IHC, IF, IP
HSP90	Enzo	ADI-SPA-840-F	9D2	1:1000	WB
Oct4	Abcam	ab19857		1:1000 (WB)<br>1:100 (IF)	WB, IF
Nanog	Cell Signaling	4903S	D73G4	1:1000	WB
Sox2	Abcam	ab97959		1:1000	WB
Hop	Enzo	ADI-SRA-1500-F	DS14F5	1:1000	WB
HIF-1α	BD	610958	54/HIF-1α	1:1000	WB
pAkt (S473)	Cell Signaling	4060S	D9E	1:1000	WB
Akt	Cell Signaling	9272S		1:1000	WB
pMEK (S217/221)	Cell Signaling	9121S		1:1000	WB
MEK	Cell Signaling	9122S		1:1000	WB
pSrc (Y416)	Cell Signaling	6943	D49G4	1:1,000	WB
Src	Cell Signaling	2109	36D10	1:1,000	WB
Actin	Santa Cruz	sc-47778	C4	1:1,000	WB
Cleaved PARP (Cl-PARP)	BD	552596	F21-852	1:1,000	WB
Cleaved Caspase-3 (Cl-Cas3)	Cell Signaling	9661S	D175	1:1000 (WB)<br>1:100 (IF)	WB, IF
His-Tag	Santa Cruz	sc-8036	H-3	1:1000	WB
Ubiquitin	Santa Cruz	sc-801t7	P4D1	1:1000	WB

HRP-conjugated goat anti-mouse IgG	GeneTex	213111-01	1:5,000	WB
HRP-conjugated goat anti-rabbit IgG	GeneTex	213110-01	1:5,000	WB
HRP-conjugated goat anti-rat IgG	Thermo Fisher Scientific	62-9520	1:5,000	WB
Alexa Fluor 488-conjugated goat anti-rabbit IgG	Thermo Fisher Scientific	A-11034	1:1,000	IF
Alexa Fluor 488-conjugated goat anti-mouse igG	Thermo Fisher Scientific	A-11001	1:1,000	IF
Alexa Fluor 594-conjugated goat anti-rabbit igG	Thermo Fisher Scientific	A-11012	1:1,000	IF
Biotinylated rabbit anti-mouse igG	Bethyl Laboratories	A90-117B	1:1,000	IHC

[1)]Application - WB: Western blot analysis; IF: immunofluorescence staining; IHC: immunohistochemistry; IP: immunoprecipitation

Table S3. Primer sequences used in this study.

Gene	Forward sequence (5´-3´)	Reverse sequence (5´-3´)
POU5F1	TGCAGCAGATCAGCCACATC	CTCGGACCACATCCTTCTCG
NANOG	CCTCCTCCATGGATCTGCTTATTCA	CAGGTCTTCACCTGTTTGTAG
SOX2	AACCAGCGCATGGACAGTTA	ATCATGCTGTAGCTGCCGTT
HSPB1	CACGCAGTCCAACGAGATCA	TTACTTGGCGGCAGTCTCAT
DNAJB1	TCATGGGTAAAGACTACTACCAGAC	GTAGGCCCGCTTGATCTCCT
HSPD1	GACACGGGCTCATTGCGG	GTGAGATGAGGAGCCAGTACC
HSPA1A	AGCTGGAGCAGGTGTGTAAC	TACCTCCTCAATGGTGGGGC
HSP90AA1	TGCTCCAAGGGTTGACATGG	TTTCTGTGCCTACGTGTGCT
HSPA4	TAAATGCTCCCTGCCTGGTG	GTGTTAATCCATGCCGTGCC
STIP1	ATGACCACTCTCAGCGTC	CTCCTTGGCTTTGTCGTA
AHSA1	CATCAGCACCCTCAAAACAG	CCCACTGGGTCTACTGACTCTC
ACTB	TCATTCCAAATATGAGATGCGTTG	TAGAGAGAAGTGGGGTGGCT
16S rRNA (for mycoplasma test)	GGGAGCAAACAGGATTAGATACCCT	TGCACCATCTGTCACTCTGTTAACCTC

Table S4. Effects of a natural product chemical library on the number of total and GFP^{high} cells in H1299/pOct4-GFP cells.

No.	Name	Total cells (% or Control)	GFP^{high} cells (% of Control)
1	acacetin	108.7	121.2
2	amentoflavone	110.5	108.7
3	amurenoside B	104.5	117.9
4	anhydroicaritin 3 -O- rhamnoside	107.5	120.2
5	apigenin	103.1	121.5
6	apigenin7-O-glucuroside	97.7	118.9
7	artemetin	78.3	48.4
8	astragalin (kaempferol - O - glucoside)	101.2	100.3
9	astrapterocarpan 3 - O - glucoside	116.9	144.6
10	avicularin (quercetin 3 -O -arabinofuranoside)	113.6	140.1
11	baicalin	117.8	144.9
12	baicalein	111.6	143.3
13	bilobetin	110.6	142.9
14	broussochalcone A	105.6	69.2
15	calycosin	83.7	75.6
16	calycosin 7 - O - glucoside	105.1	117.3
17	(+)-catechin	117.6	142.0
18	chrysin	124.6	158.3
19	clovin	116.1	176.6
20	daidzein	117.2	115.1
21	daidzin	112.8	116.0
22	7,8-dihydroxyflavone	110.8	146.2
23	4',7-dihydroxyflavone	84.4	75.3
24	diosmetin 7 - O - glucoside	93.2	92.9
25	diosmin	116.9	128.8
26	echinoisoflavanone	108.3	126.9
27	echinoisosophoranone	112.4	124.0
28	epicatechin acetate	106.3	121.5
29	(-)-epicatechin 3 - O - gallate	105.6	121.8
30	epimedin A	102.1	118.9
31	epimedin B	84.4	90.7
32	epimedin C	87.5	96.5
33	epimedoside A	92.3	77.7
34	(-)-eriodictyol	95.5	122.3
35	eupatilin	97.4	122.3
36	evodioside B	95.8	134.5
37	formononetin	85.2	92.3
38	formononetin acetate	71.2	103.9
39	galangin	96.0	108.7
40	galangin 3 - O- methyl ether	103.3	142.6
41	genistein	99.4	142.9
42	genistin	103.6	166.5
43	ginkgetin	101.9	118.4
44	hesperidin (hesperetin 7 -O-rutinoside)	99.4	93.9
45	3-hydroxyflavone	88.0	63.5
46	hyperin (hyperoside, quercetin 3 -O- galactopyranoside)	73.3	65.8
47	icariin	91.6	152.9
48	isoliquiritigenin	105.5	161.3
49	isomucronulatol 7 - O - glucoside	104.7	151.3
50	isoquercitrin	111.3	128.4
51	isorhamnetin	106.6	99.4
52	isorhamnetin 3 - O -galactoside	105.3	105.8

53	isosophhoranone	96.7	100.6
54	isoxanthohumol	74.1	62.3
55	jaceosidin	84.9	94.5
56	kaemferide	97.2	137.1
57	kaempferol	92.2	115.8
58	trifolin(kaempferol3-O-galactoside)	99.9	132.9
59	kaempferol 7-O-glucoside	93.1	114.2
60	kaempferol 3-O-(6''-coumaryl-glucosyl)(1->2) rhamnoside	89.2	134.5
61	kaempferol 3-O- glucosyl(1->2)rhamnoside	88.7	145.5
62	kaempferol 3-O- 2'', 6''-dirhamnosylglucoside	67.0	90.6
63	kaempferol 3-O-rutinoside(nicotiflorin)	87.1	89.8
64	kenusanone A	98.7	89.2
65	kenusanone C	100.9	110.5
66	kuraridin	100.7	61.6
67	linarin	104.4	108.3
68	liquiritin	93.1	138.1
69	liquiritigenin	79.2	93.0
70	liquiritigenin acetate	95.1	136.2
71	luteolin	98.2	128.6
72	luteolin 5 - O - glucoside	108.4	108.3
73	luteolin 7 - O - glucoside	98.3	141.3
74	maackiain	94.6	104.8
75	morin	99.6	157.5
76	myricetin	89.3	92.7
77	naringenin	78.4	93.3
78	naringin	99.6	94.3
79	neohesperidin	112.4	103.5
80	nepetin	109.4	140.3
81	ochnaflavone	112.3	109.2
82	oroxylin A	111.3	163.5
83	pectolinarin	103.7	134.3
84	poncirin	91.0	96.5
85	puerarin	82.2	77.8
86	psoralidin	92.5	111.1
87	quercetin	105.2	130.5
88	quercitirin	109.9	147.6
89	quercetin3-O-(6''-coumaroyl-glucosyl)(1->2)rhamnoside	115.4	108.9
90	quercetin3-O-2'',6''-dirhamnosylglucoside	114.5	138.7
91	rhoifolin	102.1	143.8
92	robinin	101.5	114.9
93	rutin	86.3	74.3
94	rutin2''-gallate	90.8	72.2
95	sciadopitysin	98.1	64.3
96	sophoraflavanone D	94.5	49.7
97	sophoraflavanone G	86.5	82.2
98	sophoraflavescenol	98.7	85.1
99	spinosin	98.9	72.2
100	6'''-feruloylspinosin	96.5	83.7
101	wogonin	87.2	73.0
102	2'''-O-syringylrutin	89.3	71.3
103	tiliroside	90.5	80.9
104	trifolirhizin	95.5	103.6
105	3',4',7'-trihydroxyflavone	95.7	70.7
106	vitexicarpin	30.8	60.7
107	vitexin	99.0	70.1
108	vitexin permethyl ether	96.8	80.9
109	swertisin acetate	86.3	57.1
110	isospinosin	92.5	51.6

111	kaempferol 3-O-4'''- acetylrhamininoside	88.7	57.7
112	kaempferol 3-O- rhamininoside	89.4	79.6
113	catharticin (alaterin, rhamnocitrin 3 -0- rhamininoside)	87.8	83.9
114	ajunol	95.7	118.7
115	aucubin	90.9	66.9
116	catalpol	91.9	62.2
117	dimethylsecologanoside	78.7	75.2
118	epivogeloside	84.4	64.5
119	campsiside	85.4	74.1
120	harpagide	81.5	56.7
121	geniposide	89.5	76.2
122	loganic acid	91.8	90.4
123	loganin	82.8	93.8
124	monotropein	73.7	62.8
125	secologanin dimethyl acetal	91.6	61.6
126	sweroside	96.2	84.1
127	aeginetoyl ajugol5''-O- b - D - quinovoside	99.3	88.1
128	6-O-(4''-O-I-a-L-rhamnosyl)vanilloylajugol	102.4	100.7
129	valeroside	96.7	98.8
130	albiflorin	98.9	87.4
131	benzoylpaeoniflorin	87.8	64.9
132	galloylpaeoniflorin	88.5	94.1
133	lactiflorin	105.4	114.8
134	oxypaeoniflorin	105.2	105.4
135	paeoniflorin	109.8	71.7
136	paeoniflorin acetate	100.1	74.2
137	1-O-beta-D-glucopyranosyl-8-O-benzoylpaeonisuffrone	108.9	115.2
138	rengyol	100.6	62.1
139	artemisinin	78.3	61.1
140	bilobalide	86.6	69.6
141	handelin	106.5	113.3
142	9-hydroxyheterogorgiolide	107.5	93.7
143	(+)-ledol	105.2	74.2
144	patulialcohol	106.3	78.2
145	aeginetic acid 5 - O - beta - D - quinovoside	102.7	68.4
146	abietic acid	95.0	118.0
147	crocin	85.2	78.0
148	tanshinone I	80.1	74.0
149	tanshinone IIA	93.8	73.3
150	ginkgolide A	97.6	115.5
151	ginkgolide B	89.0	68.4
152	ginkgolide C	90.4	86.7
153	ent-kaur-16-en-19-oic acid (kaurenoic acid)	85.7	73.1
154	taxinine	71.4	79.2
155	taxinine A	103.1	71.5
156	taxinine B	117.7	91.1
157	acacigenin B	111.3	115.6
158	acacigenin B methyl ester monoacetate	111.6	71.0
159	aleuritolic acid	102.0	89.2
160	aleuritolic acid methyl ester	106.7	95.2
161	aleuritolic acid methyl acetate	93.4	63.3
162	aleuritolic acid acetate	98.7	83.5
163	epialeuritolic acid	118.1	102.6
164	epialeuritolic acid methyl acetate	111.6	87.8

165	beta-amyrin	113.0	86.8
166	beta-amyrin acetate	119.1	96.6
167	asiatic acid	112.1	96.9
168	betulin	104.9	79.1
169	betulinic acid	98.1	70.3
170	betulinic acid methyl ester	95.0	66.9
171	corosolic acid	121.6	68.6
172	echinocystic acid	112.9	82.7
173	erythrodiol	114.6	120.6
174	esculentic acid dimethyl ester	111.7	77.5
175	esculentic acid	113.6	86.1
176	friedelin	98.7	79.9
177	hederagenin acetate	97.3	82.3
178	glabrolide	85.6	70.5
179	glycyrrhetic acid	99.5	58.8
180	glycyrrhetic acid methyl acetate	102.9	79.4
181	jaligonic acid	99.9	83.5
182	jaligonic acid dimethyl ester	97.8	62.4
183	jaligonic acid 28-monomethyl ester	100.9	62.1
184	liquiritic acid	101.4	66.7
185	syringic acid(4-hydroxy-3,5-dimethoxybenzoic acid)	78.8	53.2
186	lupeol	82.2	70.2
187	lupenone	94.2	87.1
188	mesembryanthemoidigenic acid	96.8	73.6
189	myricadiol 3 - acetate	92.2	54.8
190	30-norarjunolic acid	98.3	67.4
191	30-norhederagenin	96.1	78.7
192	syringin	91.0	57.0
193	oleanolic acid	81.8	60.4
194	oleanolic acid methyl ester	78.9	58.1
195	oleanolic acid acetate	91.1	65.4
196	oleanolic acid methyl acetate	93.0	61.5
197	oleanolic acid 3 - keto	91.8	41.6
198	panaxadiol	89.7	75.6
199	panaxatriol	97.2	58.7
200	beta-peltoboykinolic acid methyl acetate	96.7	67.4
201	phytolaccagenic acid	81.7	73.9
202	phytolaccagenin	82.2	98.0
203	phytolaccagenin triacetate	92.0	103.4
204	soyasapogenol B triacetate	95.0	71.3
205	tormentic acid	94.4	68.5
206	ursolic acid	101.5	88.2
207	3 alpha - hydroxyoleanolic acid methyl ester	98.5	78.1
208	3 beta, 21beta, 30- trihydroxyolean-12-en	95.8	65.4
209	taraxerol	86.0	77.8
210	betulafoliane diol	93.8	55.3
211	23-dihydroganoderic acid I	102.3	125.8
212	24-dihydroganoderic acid N	98.8	96.9
213	limonin	96.5	103.7
214	obacunone	97.8	99.4
215	lanosterol	96.6	108.7
216	pomolic acid 3 - acetate	81.0	98.3
217	fraxinellone	105.5	130.2
218	pomonic acid	122.6	140.9

219	isotetrahydrofraxinellone	114.3	178.6
220	hexahydrofraxinellone	118.9	230.2
221	ursolic acid	120.0	170.4
222	diosgenin	115.9	86.2
223	diosgenin acetate	117.2	179.2
224	gitogenin	113.8	156.0
225	hecogenin	129.6	139.0
226	hecogenin acetate	124.0	148.4
227	(25S)-ruscogenin	131.7	196.2
228	sarsasapogenin	133.6	164.2
229	astragaloside I	130.7	137.7
230	astragaloside II	127.8	141.5
231	astragaloside II	108.4	155.3
232	astragaloside IV	115.6	158.5
233	azukisaponin II	122.6	137.7
234	azukisaponin V	122.9	115.1
235	azukisaponin V methyl ester	131.2	134.0
236	chikusetsusaponin IV	131.3	166.7
237	ginsenoside Rg1	135.0	175.5
238	jujuboside A2	123.0	124.5
239	loniceroside A	99.9	128.9
240	narcissiflorine 6'-methyl ester	103.1	136.5
241	narcissiflorine dimethyl ester	114.3	132.1
242	niga-ichigoside F1	124.8	208.2
243	oleanolic acid 3-O-glucuroside dimethyl ester	122.4	136.5
244	oleanolic acid 3-O-(6'-methylglucuronoside)	112.3	167.3
245	sinapic acid	122.2	117.6
246	oleanolic acid 28-O-glucuronoside	117.1	171.1
247	phytolaccoside B	99.5	98.1
248	phytolaccoside E	76.9	54.4
249	phytolaccoside F	91.8	40.3
250	phytolaccoside I	101.6	71.5
251	platycodin D	100.3	65.9
252	platycoside E	94.7	96.7
253	pulsatilla saponin F	98.0	90.2
254	pulsatilla saponin H	94.7	76.7
255	14.110.	83.8	42.3
256	salsoloside C methyl ester	80.4	61.0
257	silphioside A	91.5	63.3
258	soyasaponin II methyl ester	102.9	75.4
259	stipleanoside R1 dimethyl ester	99.1	72.5
260	stipleanoside R2	105.0	54.1
261	stipleanoside R2 methyl ester	109.5	69.5
262	suavissimoside R1	95.3	61.3
263	ziyuglycoside I	85.5	89.8
264	ziyuglycoside II	79.8	47.2
265	anemarsaponin B	93.6	84.9
266	diosgegnin 3-O-rhamnosyl(1->2) glucoside	102.9	73.4
267	dioscin	21.9	5.6
268	gracillin	103.1	57.4
269	fenugreek saponin II	108.4	91.1
270	spicatoside A	101.8	56.4
271	trillin	84.7	99.0
272	americanin A	71.9	69.5

273	acetonylidene americanin A	83.7	67.5
274	americanin B	90.8	58.7
275	dimethyl lithospermic acid	99.0	89.8
276	gomisin A	94.7	87.5
277	gomisin N	95.0	62.3
278	honokiol	83.8	51.5
279	matairesinoside	96.8	122.8
280	paulownin	111.7	132.4
281	pinoresicol glucoside	114.1	147.9
282	schizandrin	113.8	152.1
283	schisantherin A (gomisin C)	102.3	107.3
284	schisantherin C	102.3	127.4
285	sesamin	104.2	94.5
286	sesamol	81.8	61.6
287	shikonin	82.5	116.0
288	sesamolin	115.0	132.9
289	sesangolin	103.2	107.8
290	silybin	109.0	134.2
291	simplexoside	110.2	129.2
292	magnolol	109.1	101.8
293	angelican	119.2	163.9
294	bergapten	93.1	119.6
295	bergaptol acetate	97.5	125.1
296	coumarin	118.0	93.2
297	6-methyl coumarin	107.7	125.1
298	decursin	107.5	83.6
299	decursinol	117.6	84.0
300	esculetin	106.1	113.2
301	esculin	74.9	184.9
302	glabralactone	87.6	108.2
303	imperatorin	101.8	101.8
304	isoimperatorin	114.2	144.7
305	isooxypeucedanin	100.4	102.3
306	trans-khellactone	97.0	82.2
307	nodakenetin	96.8	77.2
308	modakenin	101.9	139.3
309	osthol	82.7	65.3
310	evodiamine	22.4	42.1
311	oxypeucedanin	79.9	76.0
312	oxypeucedanin hydrate	94.7	106.3
313	oxypeucedanin methanolate	98.1	74.8
314	pabulenol	87.3	61.4
315	prangolarin[(+)-oxypeucedanin]	87.8	66.1
316	psoralen	106.4	113.0
317	scopolin	70.9	79.9
318	umbelliferone	72.0	87.0
319	shikimic acid	100.8	107.9
320	xanthotoxin(8-methoxypsoralen)	102.4	91.0
321	trans - resveratrol	89.6	72.0
322	N-acetylanonaine[(-)-acetylanonaine]	108.3	106.7
323	N-acetylanthranilic acid methyl ester	103.9	119.3
324	aconitine	104.2	59.8
325	adenosine	90.3	91.7
326	ajmalicine	79.9	66.9

327	allantoin	105.9	90.5
328	amygdaline	106.4	101.2
329	berberine-HCl	107.0	104.3
330	L--citrulline	112.3	93.3
331	confusameline	106.3	109.1
332	crassicauline A	102.1	88.2
333	dehydroevodiamine-HCl	89.7	66.9
334	3-deoxyhokbusine A	83.9	114.2
335	dictamine	98.2	103.5
336	evolitrine	108.4	100.8
337	gamma-fagarine	98.9	81.9
338	harmaline(dihydroharmine)	100.9	87.4
339	6-hydroxykynurenic acid	85.6	103.6
340	hypaconitine	88.3	68.3
341	indole 3 -butyric acid	104.6	94.3
342	kokusaginine	104.4	71.0
343	lycoctonine	95.6	41.7
344	magnoflorine	98.6	63.3
345	matrine	95.9	47.3
346	neoline	98.0	67.0
347	nicotinamide	80.8	42.7
348	nicotinic acid	79.6	43.3
349	oxymatrine	97.8	51.0
350	palmatine	97.8	83.7
351	piperine	105.8	69.3
352	prunasine	93.8	49.7
353	ricinine	91.6	75.0
354	N-demethylricinine	75.2	40.7
355	robustine	81.7	51.3
356	rutaecarpine	89.4	79.7
357	skimmianine	87.1	30.3
358	sophocarpine	94.5	56.3
359	synephrine	96.3	65.7
360	uridine	103.7	54.0
361	acteoside	86.5	62.0
362	androsin	73.9	38.7
363	arbutin	82.9	62.7
364	salicyclic acid	82.7	30.7
365	angeliticin A	86.5	58.0
366	benzoic acid	89.4	78.0
367	caffeic acid(3,4-dihydroxycinnamin acid)	86.2	55.0
368	caffeic acid methyl ester	85.6	93.7
369	caffeic aldehyde	72.6	43.7
370	chlorogenic acid	59.9	49.3
371	coniferin	112.4	101.5
372	corilagin	124.3	110.7
373	3,5-dicaffeoylquinic acid methyl ester	118.6	68.0
374	2,5-dihydroxyacetophenone	121.9	47.6
375	gentisic acid	119.9	93.2
376	emodin	110.8	116.0
377	emodin8-O-glucoside	118.6	71.4
378	ferulic acid	108.7	96.1
379	gallic acid	105.8	129.6
380	gallic acid methyl ester	133.0	116.5

381	gallic aldehyde	133.1	78.6
382	vanillic acid(3-methoxy-4-hydroxybenzoic acid)	131.3	156.3
383	1-O-galloyl beta-D-glucose	144.2	171.4
384	gastrodin	120.8	148.1
385	geraniin	128.8	131.1
386	hexadecanoylferulate	100.4	96.6
387	homoarbutin	110.9	89.3
388	p-hydroxybenzoic acid	124.7	109.2
389	4-hydroxybenzylmethyl ether	129.1	124.8
390	hydroxyprenylhomoarbutin	128.0	117.0
391	isoferulic acid	140.9	125.7
392	khellin	125.5	135.9
393	lawsone	130.8	138.8
394	mangiferin	104.3	95.6
395	paeonol	111.2	112.1
396	paeonoside	117.2	114.1
397	pentagalloyl beta-D-glucose	125.9	133.5
398	piperic acid methyl ester	126.7	146.1
399	pirolatin	125.5	124.8
400	protocatechualdehyde(3,4-dihydroxybenzaldehyde)	116.4	121.4
401	protocatechuic acid(3,4-dihydroxybenzoic acid)	96.8	87.9
402	salicin	100.5	138.7
403	4-hydroxy-3-methoxycinnamaldehyde	120.0	119.0
404	vanillin(4-hydroxy-3-methoxybenzaldehyde)	109.8	87.5
405	2,4-dihydroxybenzoic acid	102.2	101.2
406	3,4-methoxybenzoic acid	108.9	127.4
407	3,5-dihydroxybenzoic acid	108.2	133.9
408	cis-4-hydroxycinnamic acid	105.7	114.9
409	1,4-dihydroxy-2-naphthoic acid	80.7	101.2
410	cyasterone	102.7	106.5
411	ecdysterone	112.5	104.8
412	ergosterol	132.8	139.9
413	5-dihydroergosterol	114.7	140.5
414	fucosterol	122.6	108.9
415	pokeseed ccerebroside	119.0	138.7
416	beta-sitosterol3-O-glucoside	101.4	144.0
417	6'-palmitoyl-beta-sitosterol3-O-glucoside	100.8	117.3
418	alpha-spinasterol	109.6	139.9
419	17- hydroxy-12-O-benzoyllineolon	122.5	132.7
420	12-O-benzoyllineolon	130.3	135.7
421	aralia cerebroside	127.0	145.2
422	aralia ceramide	129.8	128.0
423	beta-sitosterol	121.6	128.6
424	soya-cerebroside I	121.3	105.4
425	jio-cerebroside(soyacerebroside I & II)	96.3	151.8
426	azelaic acid	123.2	113.7
427	5-(alpha-D-galactopyranosyloxymethl)-2-furancarboxaldehyde	113.4	112.5
428	1-monopalmitoyl-rac-glycerol(monopalmitin)	119.3	137.5
429	D-mannitol	122.8	139.9
430	eleutheroside C (ethyl alpha-D-galactopyranoside)	120.6	141.7
431	(1R,3R,4R,5R)-(-)-quinic acid	112.0	131.0
432	tiglic acid	96.3	140.5

Table S5. Tumor initiating cell frequency of each group calculated using ELDA software.

Cell line used for tumor xenograft model	Group	Tumor initiating cell frequency			Fold change	P value
		Lower	Estimate	Upper		
H460	Control	1/311	1/109	1/38.2	128.5	1.27×10^{-10}
H460	Evodiamine	1/55703	1/14002	1/3519.9		
MDA-MB-231	Control	1/128	1/46	1/16.8	61.8	4.9×10^{-9}
MDA-MB-231	Evodiamine	1/6870	1/2843	1/1176.5		

Table S6. IC_{50} values of evodiamine against cancer cell viability.

Cell line	IC_{50} (μM)
H1299	2.35
A549	0.73
H460	0.87
H226B	1.97
HCT116	1.29
MDA-MB-231 (MDA-231)	1.18

Table S7. Synergistic effect of the combinatorial treatment between evodiamine and chemotherapeutic drugs.

Experiment	Treatment A	Treatment B	Expected[1)]	Observed	Index[2)]
MTT assay	Evodiamine 5 μM	Carboplatin 50 μM	0.337	0.267	1.26
		Carboplatin 100 μM	0.326	0.260	1.25
		Carboplatin 200 μM	0.285	0.263	1.08
MTT assay	Evodiamine 5 μM	Paclitaxel 5 nM	0.358	0.227	1.58
		Paclitaxel 10 nM	0.342	0.250	1.37
		Paclitaxel 20 nM	0.308	0.256	1.20
Colony formation assay	Evodiamine 0.5 μM	Carboplatin 10 μM	0.134	0.067	2.02
		Paclitaxel 10 nM	0.085	0.013	6.40

[1)]Expected: growth inhibition rate of treatment A x growth inhibition rate of treatment B
[2)]Index: Expected growth inhibition rate / Observed growth inhibition rate

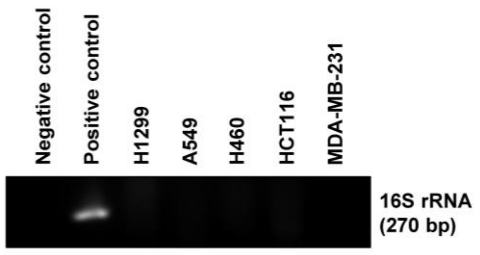

Figure S1. A representative result from analysis of mycoplasma contamination in cancer cells.

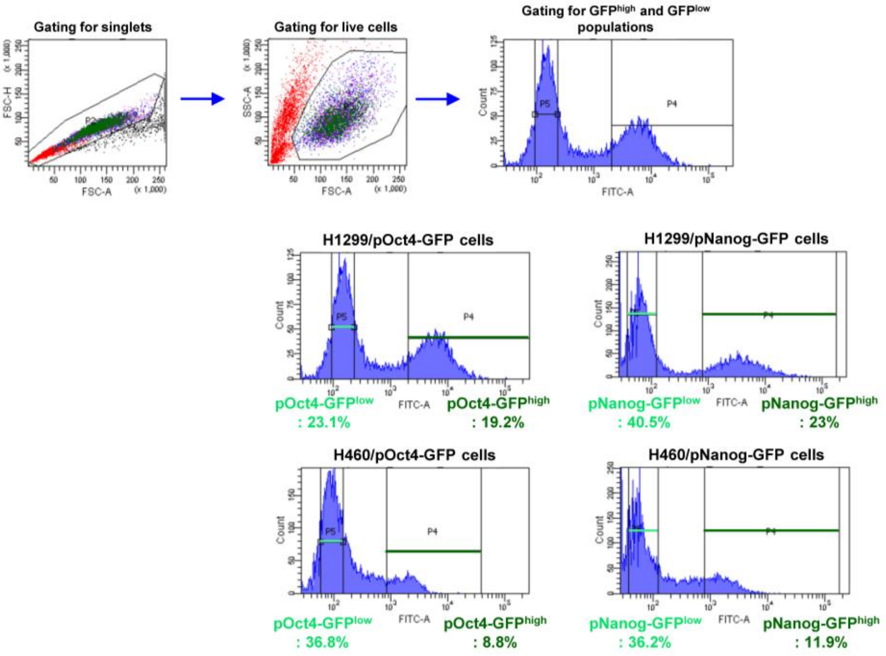

Figure S2. Gating strategy to isolate GFP^{high} and GFP^{low} populations.

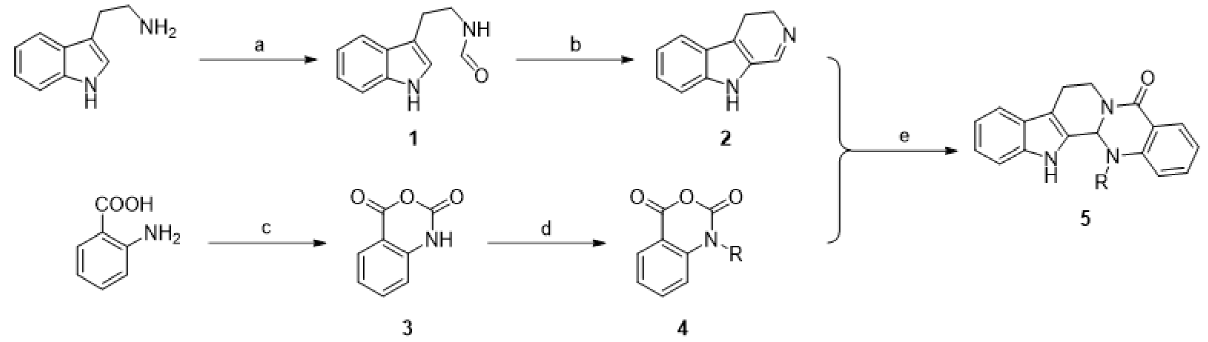

Figure S3. Synthetic scheme for the synthesis of evodiamine (Evo). Reagents and conditions: (a) Ethyl formate, 80°C, 6 h; (b) $POCl_3$, CH_2Cl_2, 0°C, 4 h; (c) Triphosgene, THF, 50°C, 3 h; (d) Iodomethane, DIPEA, DMAc, 40°C, overnight

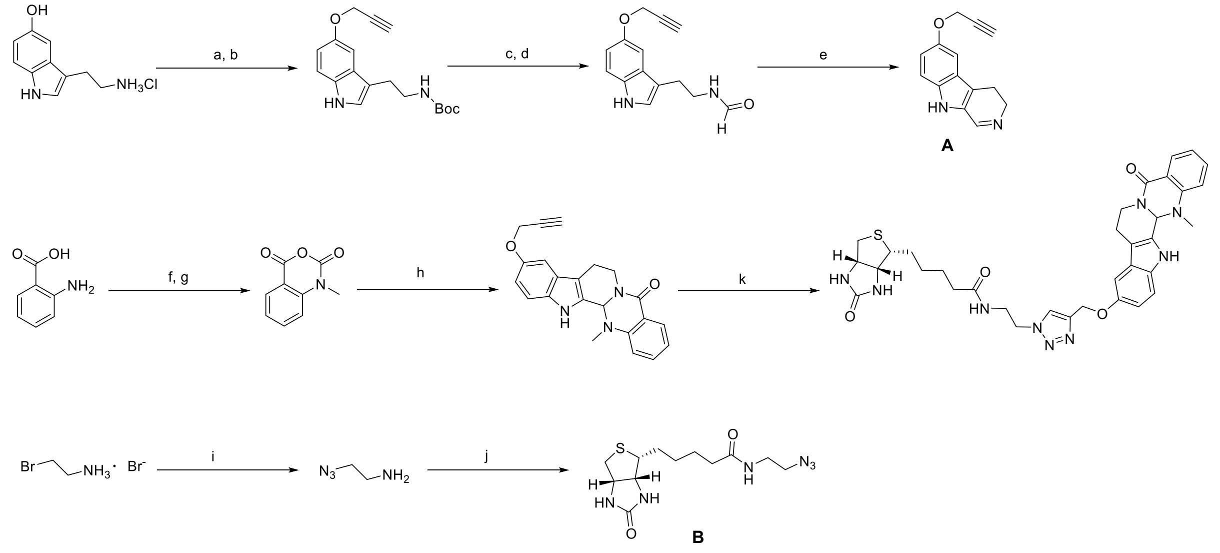

Figure S4. Synthetic scheme for the synthesis of biotinylated evodiamine (biotinylated Evo). Reagents and conditions: (a) $(Boc)_2O$, TEA, THF, 0°C to rt, 99%; (b) Propargyl bromide, K_2CO_3, DMF, 0°C to rt, 56%; (c) TFA, DCM, 0°C to rt, 98%; (d) Ethyl formate, 80°C, 56%; (e) $POCl_3$, DCM, 0°C to rt, 81%; (f) Triphosgene, THF, 50°C, 95%; (g) DIPEA, MeI, DMAc, 0°C to 40°C, 76%; (h) **A**, DCM, 50°C, 66%; (i) NaN_3, KOH, DW, 45°C, 77%; (j) N-succinimidyl D-Biotinate, TEA, MeOH, 33°C, 97%; (k) **B**, Sodium L-Ascorbate, $CuSO_4$, ^tBuOH, DW, rt, 44%

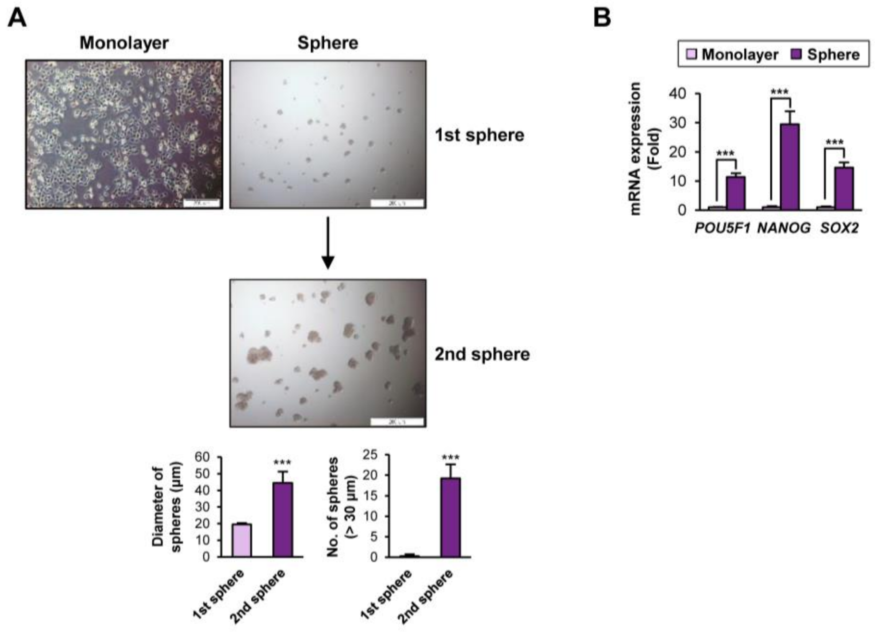

Figure S5. Stemness characteristics of H1299 cells grown in spheres compared with those grown in monolayer. (**A**) Differences in morphology (Top) and sphere formation capacity between H1299 cells grown in monolayer and those grown in sphere. (**B**) Changes in the mRNA expression of stemness markers (*POU5F1*, *NANOG*, and *SOX2*) in H1299 cells grown in sphere by comparison with those grown in monolayer. The bars represent the mean ± SD; ****P* < 0.001, as determined by a two-tailed Student's *t*-test by comparison with the corresponding control group. Scale bars: 200 μm.

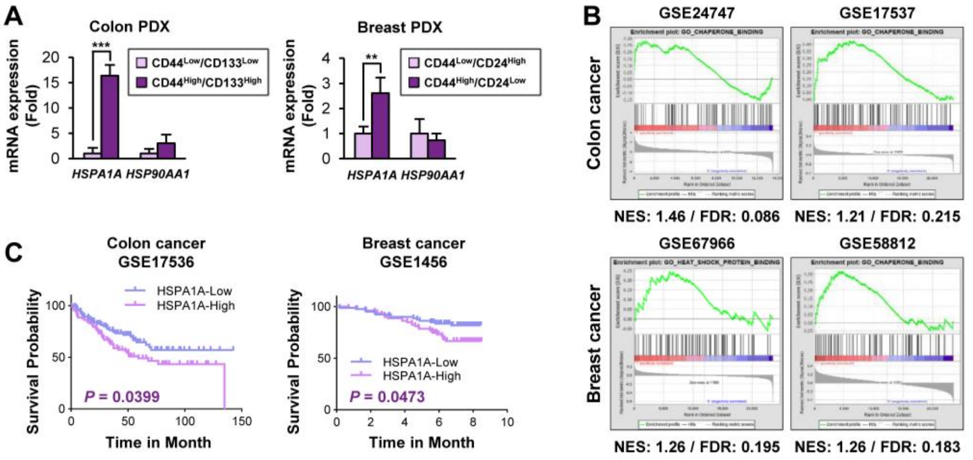

Figure S6. Regulation of *HSPA1A* expression in putative cancer stem cells isolated from colon and breast cancer patients-derived tumor cells and the association of *HSPA1A* expression with the HSP system and prognosis in patients with colon and breast cancer. (**A**) The *HSPA1A* and *HSP90AA1* mRNA expression in putative cancer stem cells isolated from colon and breast cancer patients-derived tumor cells was analyzed by real-time PCR. (**B**) GSEA of publicly available data for the HSP system-related gene sets in colon and breast cancer. (**C**) Kaplan-Meier survival analysis for the association of *HSPA1A* expression with overall survival of patients with colon and breast cancer. The bars represent the mean ± SD; $^{**}P < 0.01$ and $^{***}P < 0.001$, as determined by a two-tailed Student's *t*-test by comparison with the corresponding control group.

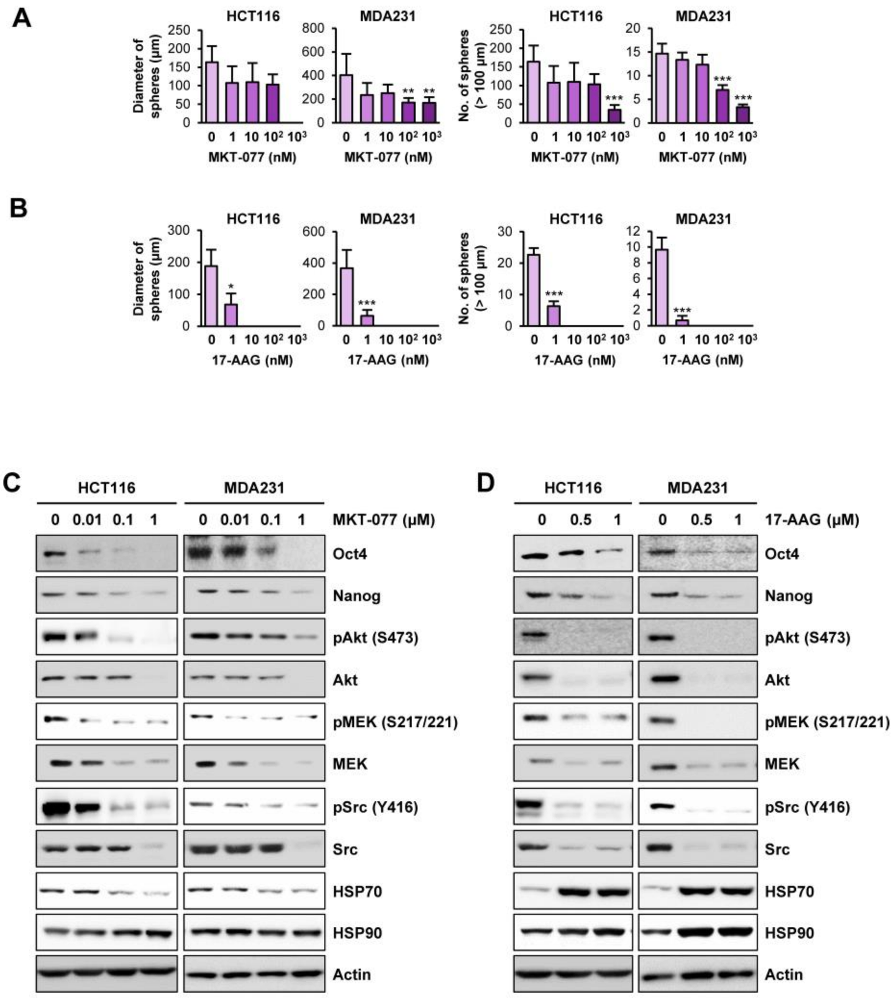

Figure S7. Regulation of sphere formation and the expression of stemness markers and the HSP70/HSP90 client proteins by treatment with HSP70 and HSP90 inhibitors in human colon and breast cancer cells. (**A-D**) Effects of pharmacological inhibitors of HSP70 (MKT-077) and HSP90 (17-AAG) on sphere formation (**A, B**) and protein expression of CSC markers and clients of the HSP system (**C, D**) in HCT116 and MDA-MB-231 (MDA231) cells grown in normal adherent conditions, as determined by the sphere formation assay (**A, B**) and Western blot analysis (**C, D**). The bars represent the mean ± SD; *P < 0.05, **P < 0.01, and ***P < 0.001, as determined by a two-tailed Student's t-test by comparison with the control group.

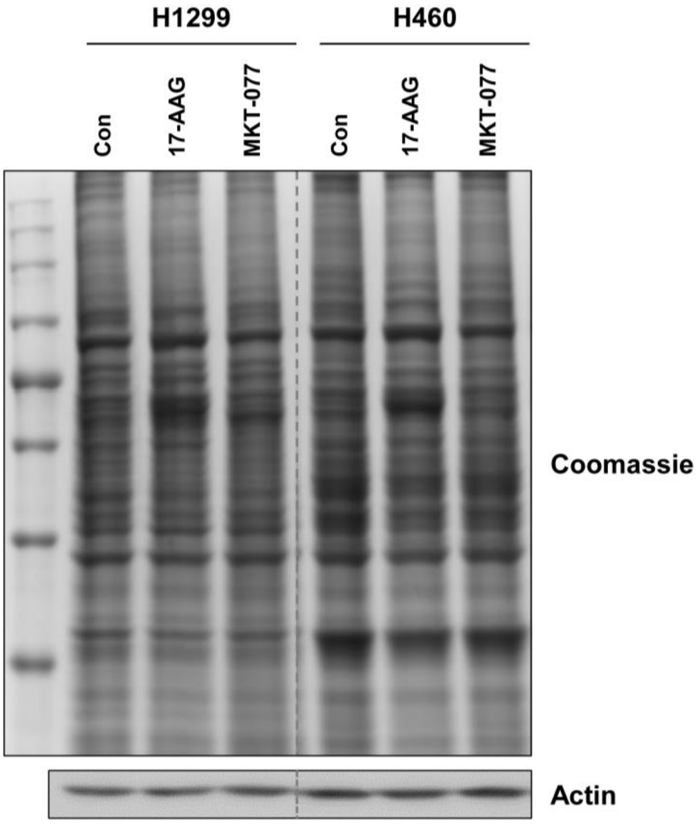

Figure S8. Changes in the global protein expression by treatment with 17-AAG and MKT-077. H1299 and H460 cells were treated with the indicated compounds for 2 days. Cell lysates were separated by SDS-PAGE, followed by Coomassie brilliant blue staining.

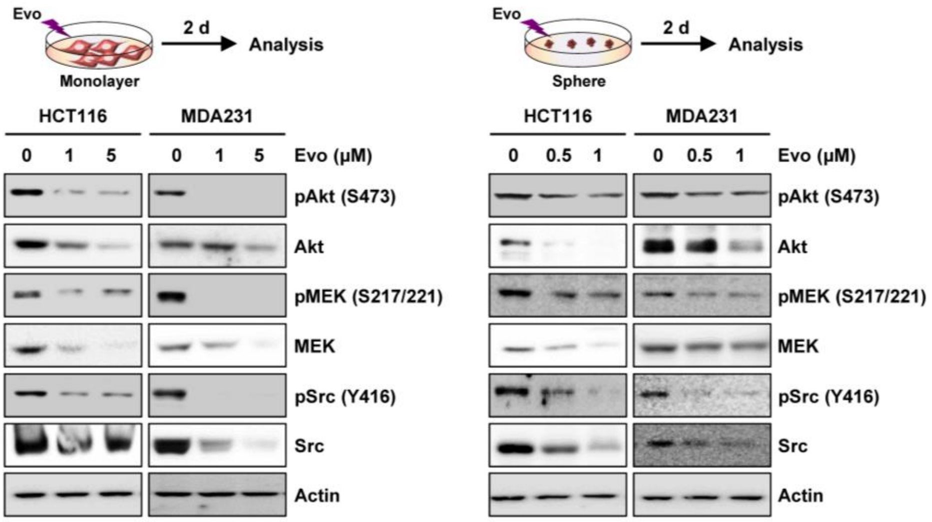

Figure S9. Regulation of total and phosphorylated forms of HSP70/HSP90 client proteins by treatment with evodiamine in human colon and breast cancer cells. Effects of evodiamine (Evo) on the expression of Akt, MEK, and Src and their phosphorylated forms in HCT116 and MDA-MB-231 (MDA231) cells grown in monolayer (left) or sphere-forming (right) conditions were determined by Western blot analysis.

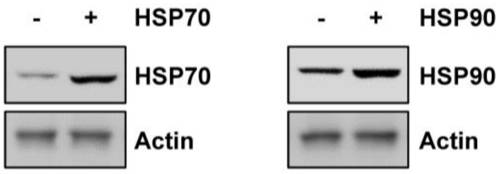

Figure S10. Regulation of HSP70 and HSP90 expression in H1299 cells transfected with HSP70 or HSP90 expression vectors. The protein expression of HSP70 and HSP90 in H1299 cells carrying ectopically overexpressed HSP70 (left) and HSP90 (right) was determined by Western blot analysis.

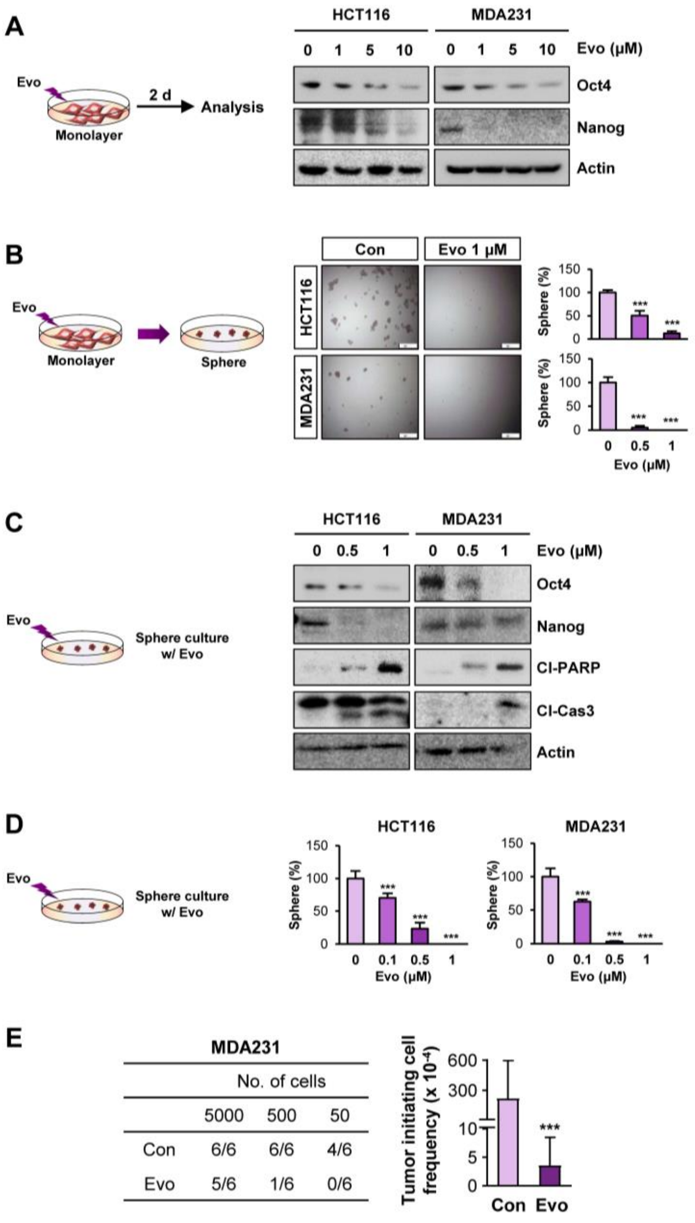

Figure S11. Effects of evodiamine on the functional features of stem cells of human colon and breast cancer cells. (**A, C**) The protein expression of stemness markers (**A, C**) and cleaved forms of PARP and caspase-3 (Cl-PARP and Cl-Cas3) (**C**) in evodiamine (Evo)-treated HCT116 and MDA-MB-231 (MDA231) cells grown in monolayer (**A**) and sphere (**C**) was determined by Western blot analysis. (**B, D**) Effects of evodiamine on sphere formation capacity of HCT116 and MDA-MB-231 cells were determined by sphere formation assay. (**E**) The effect of evodiamine on the tumorigenicity of MDA-MB-231 cells was determined by limiting dilution assay. Tumor initiating cell frequency was determined by ELDA. The bars represent the mean ± SD; ****P* < 0.001, as determined by a two-tailed Student's t-test by comparison with the control (Con) group. Scale bars: 500 μm (**B**).

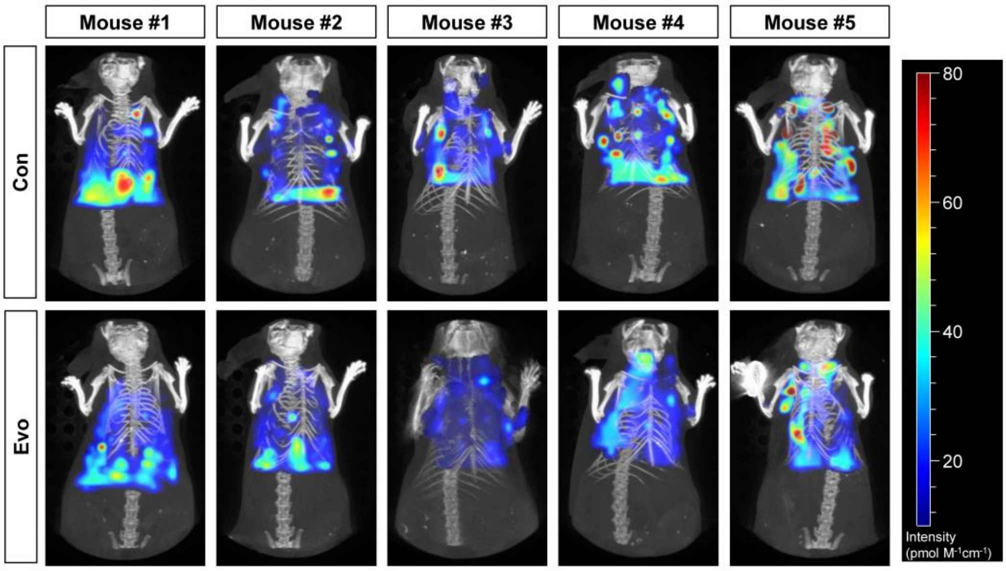

Figure S12. Bioluminescence images showing the effect of evodiamine on *Kras*$^{G12D/+}$-driven lung tumorigenesis. Representative bioluminescence images are shown in **Figure 6A**.

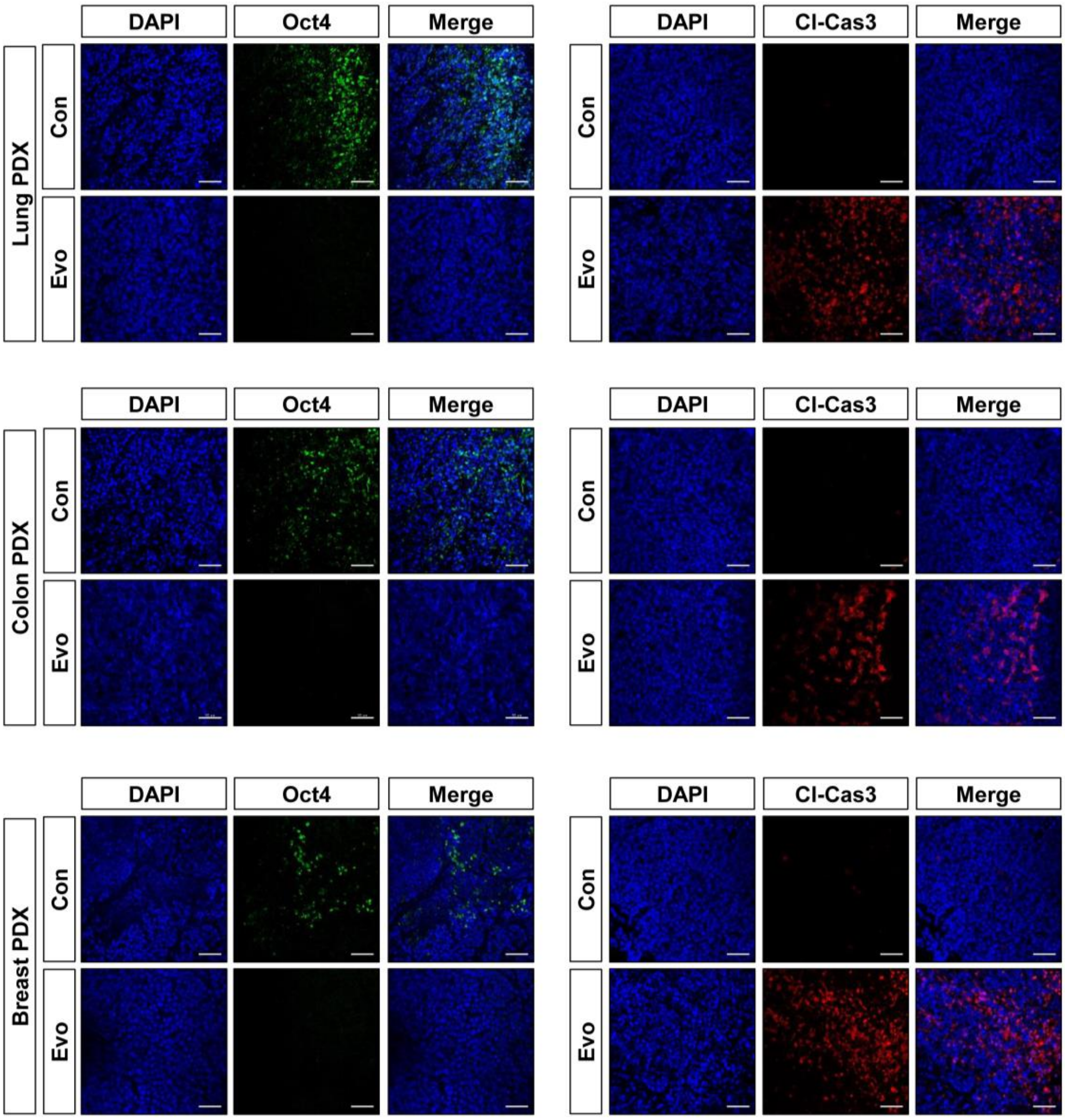

Figure S13. Representative immunofluorescence images showing the effect of evodiamine on the expression of Oct4 and cleavage of caspase-3 in tumor tissues derived from lung, colon, and breast PDX models. Quantitative analyses of the immunofluorescence images are shown in **Figure 6G**. Cl-Cas3: cleaved caspase-3. Scale bars: 50 μm.

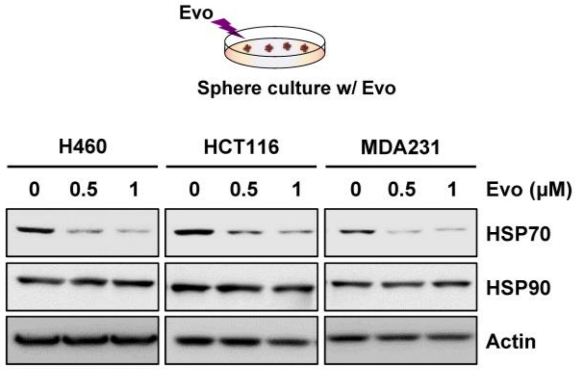

Figure S14. Effect of evodiamine on the expression of HSP70 and HSP90 in cells grown in sphere. The protein expression of HSP70 and HSP90 in evodiamine (Evo)-treated H460, HCT116, and MDA-MB-231 (MDA231) cells grown in sphere was determined by Western blot analysis.

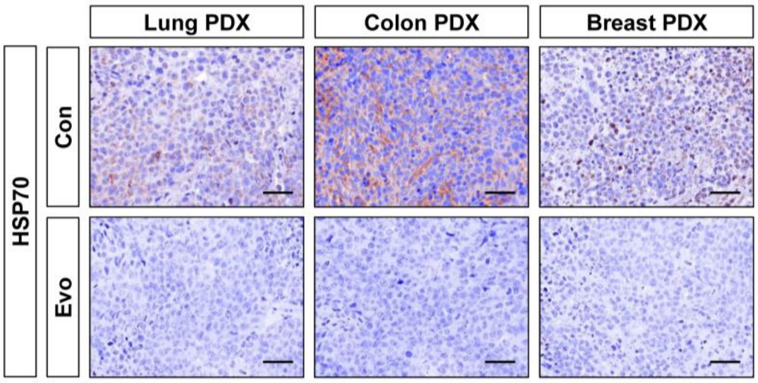

Figure S15. Representative immunohistochemistry images showing the effect of evodiamine on the expression of HSP70 in tumor tissues derived from lung, colon, and breast PDX models. Quantitative analyses of the immunohistochemistry images are shown in **Figure 7D**. Scale bars: 500 μm.

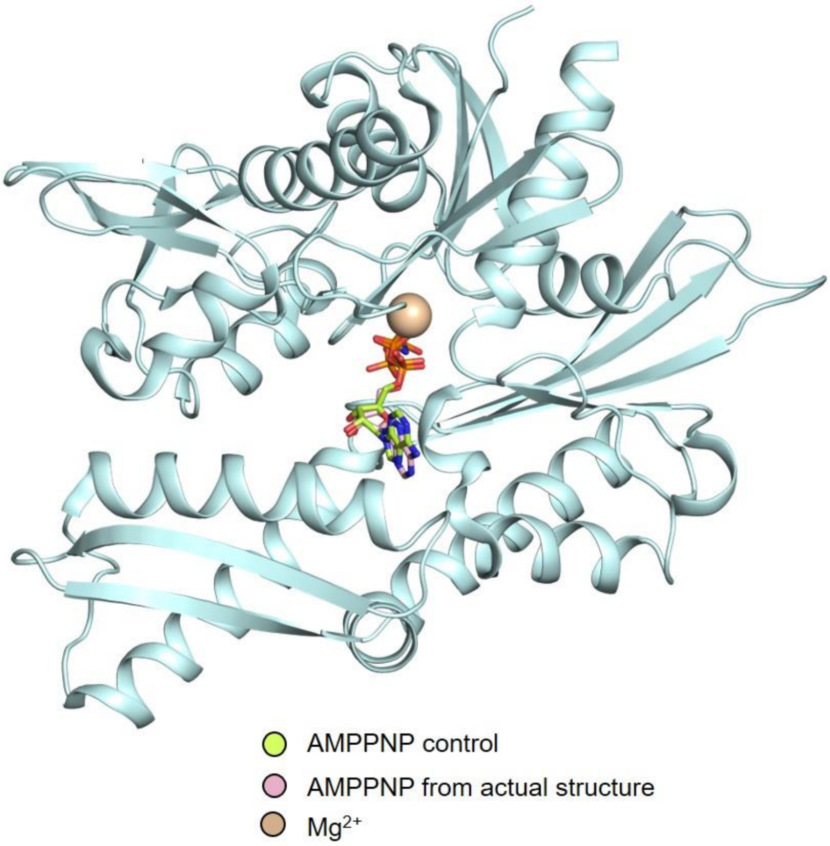

Figure S16. The control docking experiment result of the Hsp70 with AMP-PNP. The AMP-PNP in the complex structure of Hsp70 (PDB ID: 2E8A) and docking model are shown as pink and yellow green models, respectively. Mg^{2+} ion was adapted from the crystal structure of Hsp70 in complex with AMPPINP (PDB ID: 2E8A) coloured in brown. Oxygen atoms is denoted red.

Appendix 1

Raw data of STR DNA profiles for human cancer cell lines

1. Results summary

Sample	Amelogenin	D8S1179	D21S11	D7S820	CSF1PO	D3S1358	TH01	D13S317	D16S539	D2S1338	D19S433	vWA	TPOX	D18S51	D5S818	FGA
H1299	XX	10, 13	32.2	10	12	17	6, 9.3	12	12, 13	23, 24	14	16, 18	8	16	11	20
H460	XY	12	30	9, 12	11, 12	15, 18	9.3	13	9	17, 25	14	17	8	13, 15	9, 10	21, 23
A549	XY	13, 14	29	8, 11	10, 12	16	8, 9.3	11	11, 12	24	13	14	8, 11	14, 17	11	23
HCT116	XX	10, 14, 15	29, 30	11, 12	7, 10	12, 16, 17	8, 9	10, 12	11, 13	16	11, 12	17, 22	8, 9	15, 17	10, 11	18, 23
MDA MB231	XX	13	30, 33.2	8	12, 13	16	7, 9.3	13	12	21	11, 14	15	8, 9	11, 16	12	22, 23

2. Raw data

(1) H1299

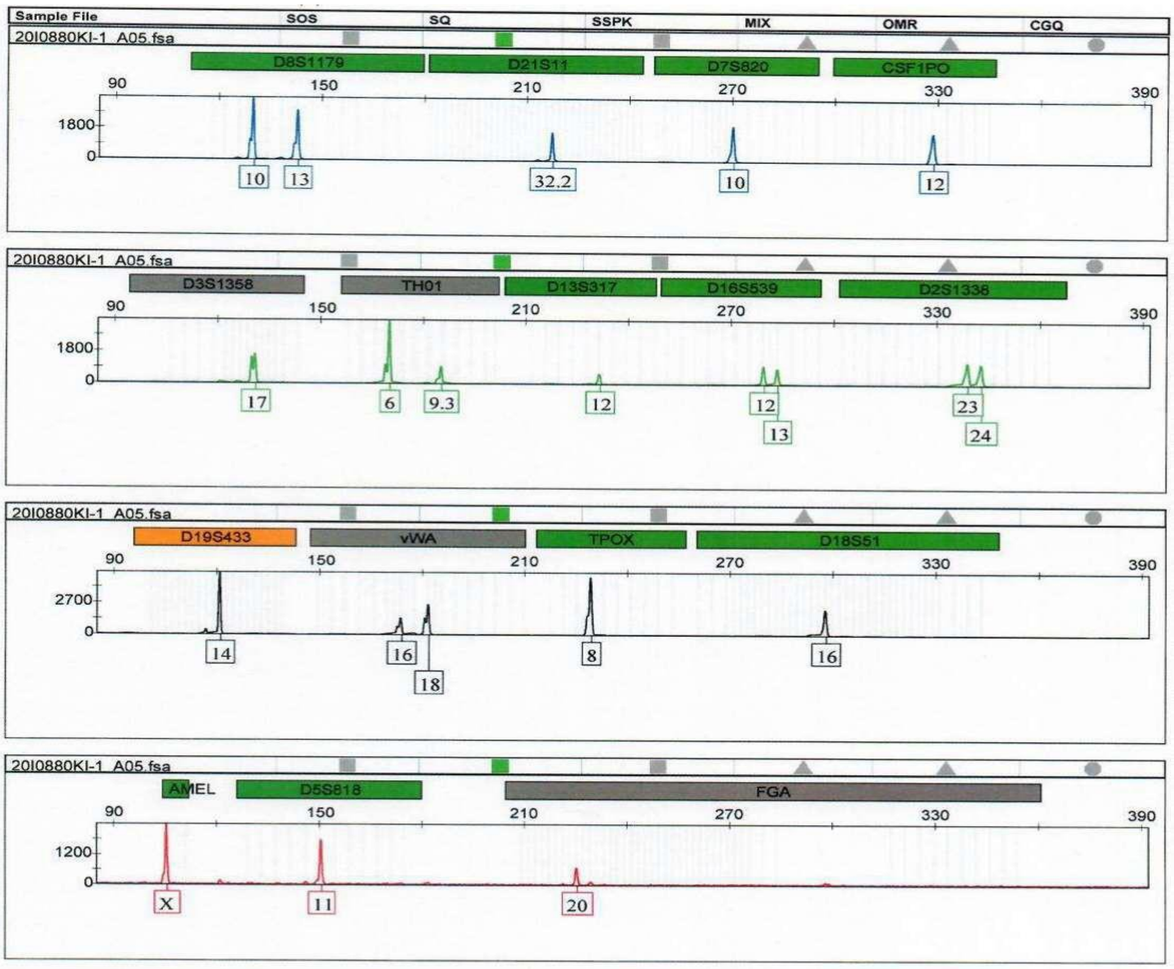

(2) H460

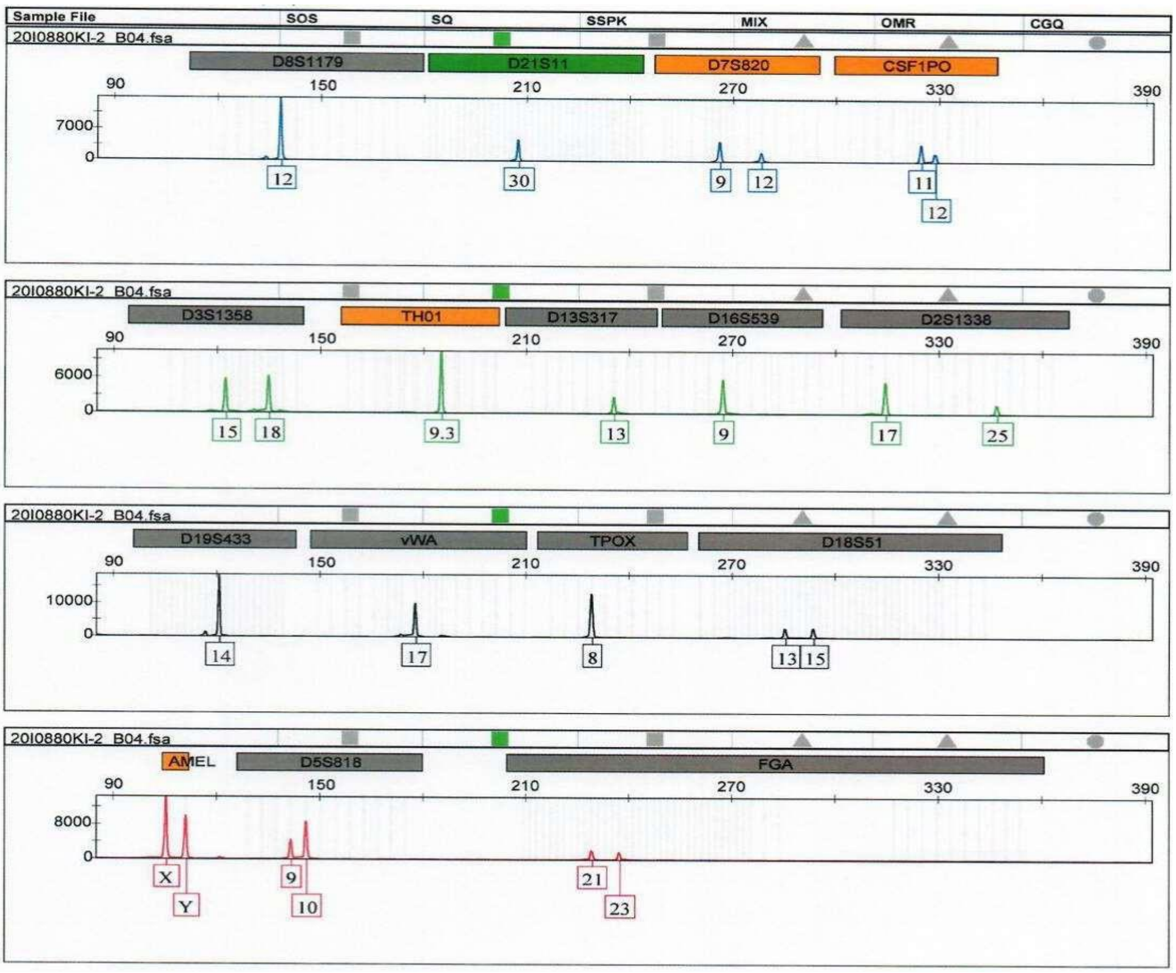

Sample File
SOS
SQ
SSPK
MIX
OMR
CGQ
2010880KI-2_B04.fsa
D8S1179
D21S11
D7S820
CSF1PO
12
30
9
12
11
12
D3S1358
TH01
D13S317
D16S539
D2S1338
15
18
9.3
13
9
17
25
D19S433
vWA
TPOX
D18S51
14
17
8
13
15
AMEL
D5S818
FGA
X
Y
9
10
21
23

(3) A549

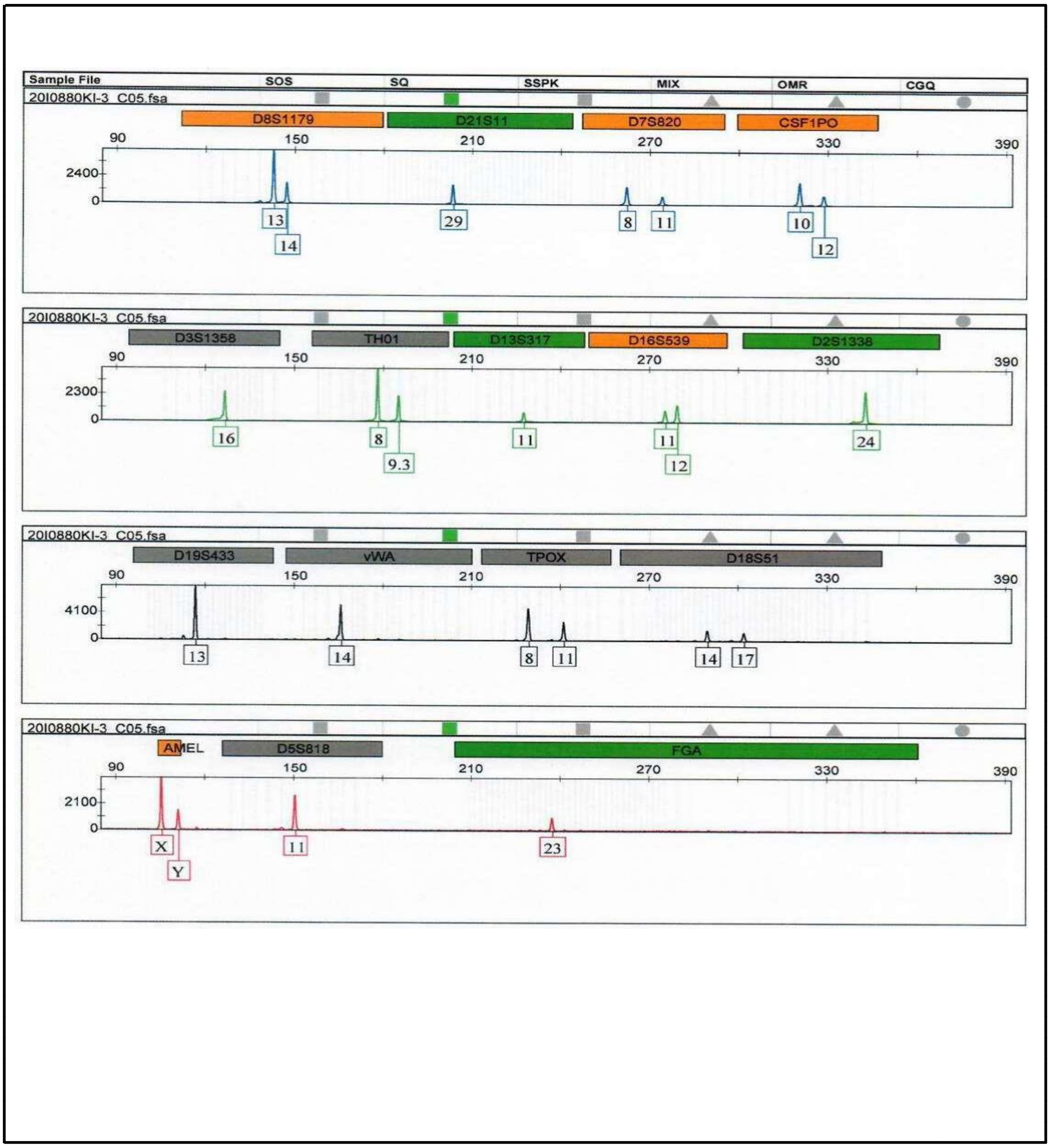
Sample File
SOS
SQ
SSPK
MIX
OMR
CGQ
2010880KI-3_C05.fsa
D8S1179
D21S11
D7S820
CSF1PO
13
14
29
8
11
10
12
D3S1358
TH01
D13S317
D16S539
D2S1338
16
8
9.3
11
11
12
24
D19S433
vWA
TPOX
D18S51
13
14
8
11
14
17
AMEL
D5S818
FGA
X
Y
11
23

(4) HCT116

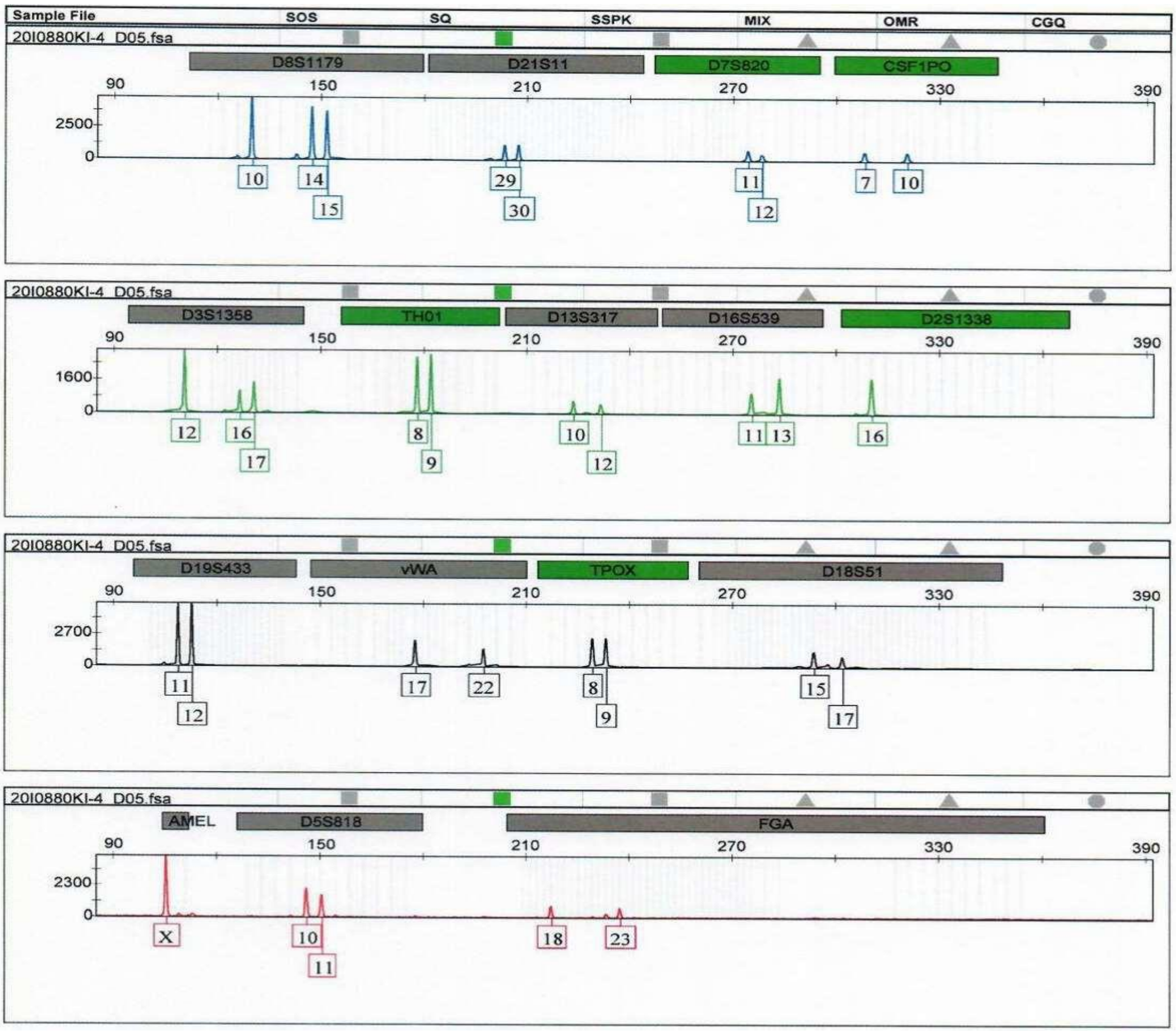

Sample File
SOS
SQ
SSPK
MIX
OMR
CGQ
2010880KI-4 D05.fsa
D8S1179
D21S11
D7S820
CSF1PO
90
150
210
270
330
390
2500
0
10
14
15
29
30
11
12
7
10
2010880KI-4 D05.fsa
D3S1358
TH01
D13S317
D16S539
D2S1338
1600
0
12
16
17
8
9
10
12
11
13
16
2010880KI-4 D05.fsa
D19S433
vWA
TPOX
D18S51
2700
0
11
12
17
22
8
9
15
17
2010880KI-4 D05.fsa
AMEL
D5S818
FGA
2300
0
X
10
11
18
23

(5) MDA-MB-231

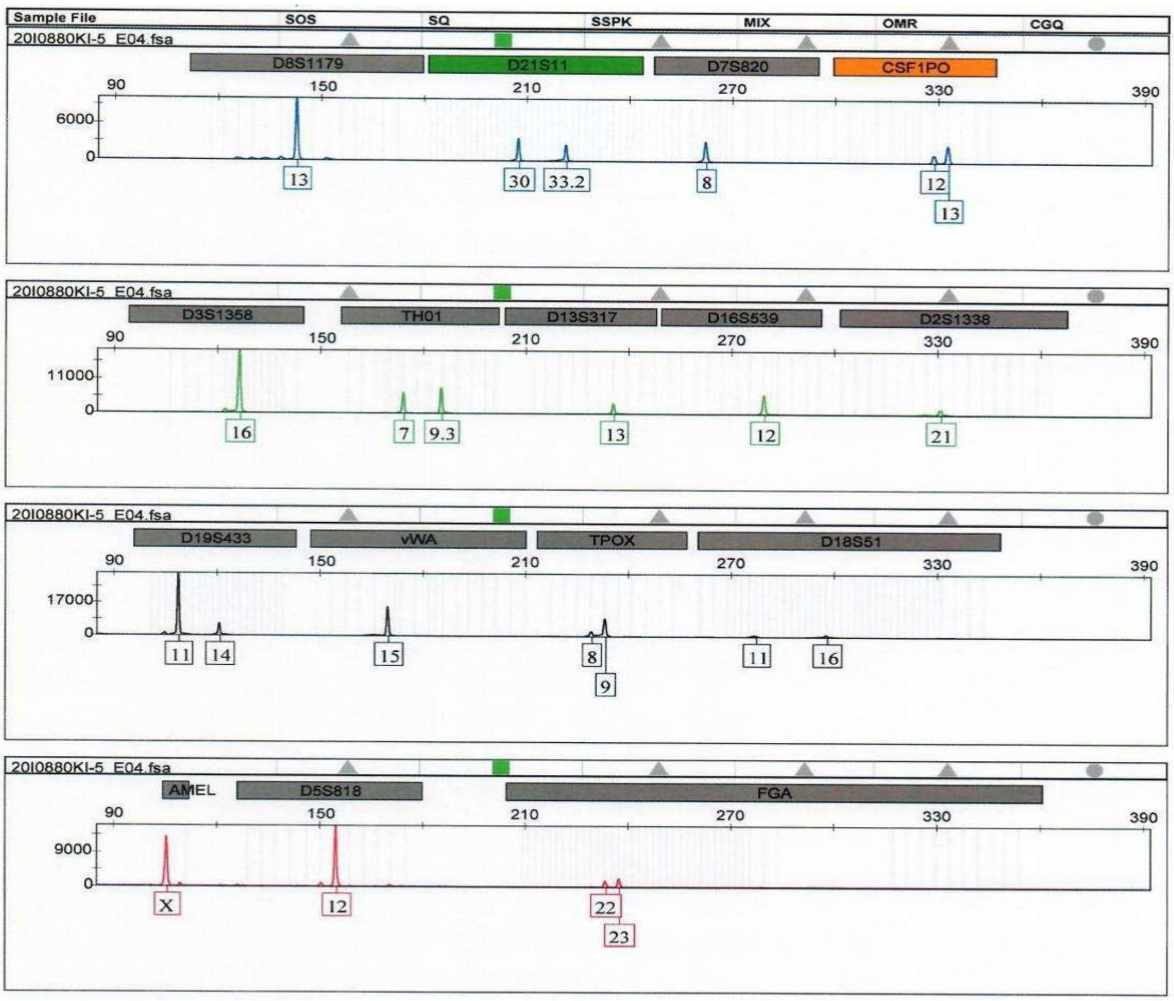

Sample File
SOS
SQ
SSPK
MIX
OMR
CGQ
2010880KI-5_E04.fsa
D8S1179
D21S11
D7S820
CSF1PO
13
30
33.2
8
12
13
D3S1358
TH01
D13S317
D16S539
D2S1338
16
7
9.3
13
12
21
D19S433
vWA
TPOX
D18S51
11
14
15
8
9
11
16
AMEL
D5S818
FGA
X
12
22
23

Appendix 2

Comparison of ^{1}H-NMR spectra between synthesized and commercial evodiamine

Synthesized Evodiamine (400 MHz, *d*-DMSO)

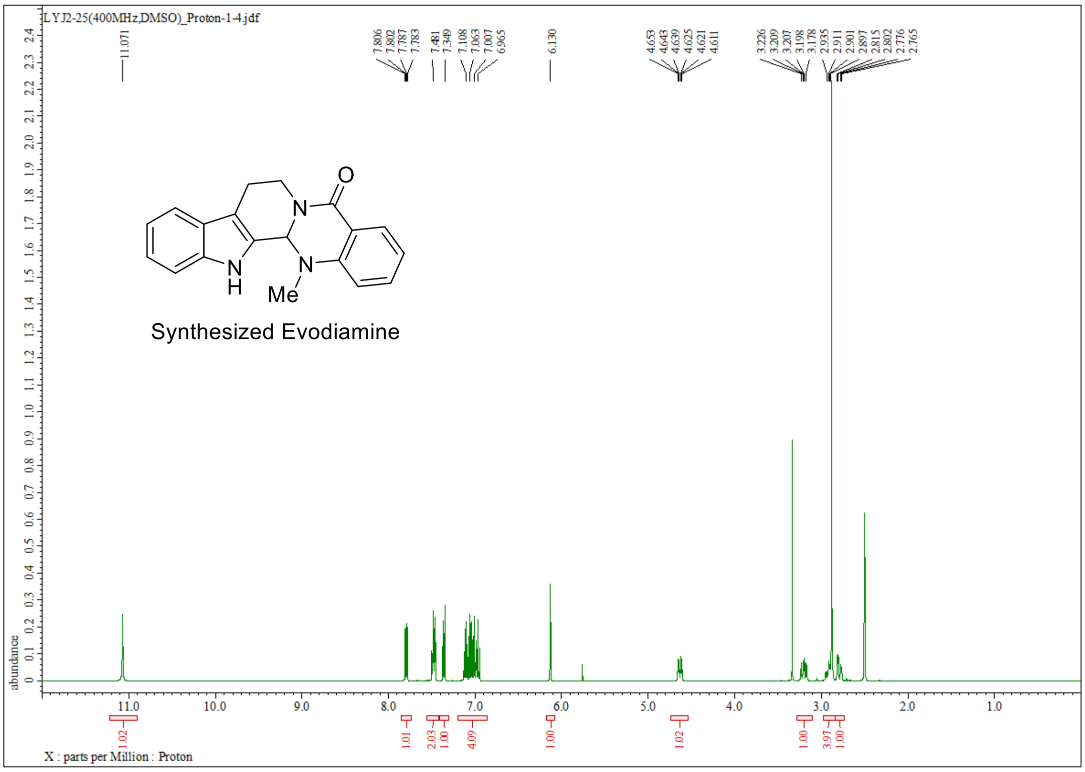

Commercial Evodiamine (400 MHz, *d*-DMSO)

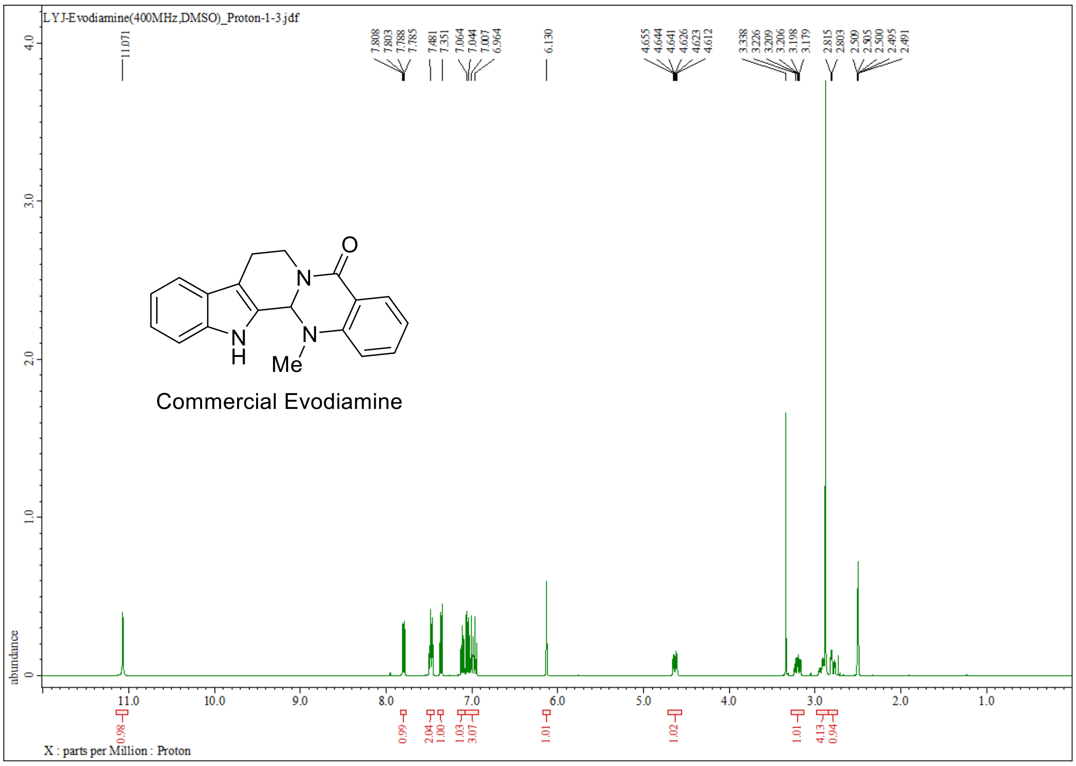

Supplement: Supplementary file 1 — Supplementary figures and tables, appendices. [file thnov11p2932s1.pdf]
